# Elucidation of the Sodium-Ion Storage Behaviors in Hard Carbon Anodes through Pore Architecture Engineering

**DOI:** 10.1021/acsnano.5c03700

**Published:** 2025-06-09

**Authors:** Wenbin Jian, Xueqing Qiu, Huaican Chen, Jian Yin, Wen Yin, Husam N. Alshareef, Wenli Zhang

**Affiliations:** † Guangdong Provincial Key Laboratory of Plant Resources Biorefinery, School of Chemical Engineering and Light Industry, 47870 Guangdong University of Technology (GDUT), 100 Waihuan Xi Road, Panyu District, Guangzhou 510006, China; ‡ Guangdong Provincial Laboratory of Chemistry and Fine Chemical Engineering Jieyang Center, Jieyang 515200, China; § Center for Renewable Energy and Storage Technologies (CREST), 127355 King Abdullah University of Science and Technology (KAUST), Thuwal 23955-6900, Saudi Arabia; ∥ Laboratory of Environmental Sciences and Technology, Xinjiang Technical Institute of Physics & Chemistry, 12381 Chinese Academy of Sciences, Urumqi 830011, China; ⊥ Institute of High Energy Physics, Chinese Academy of Sciences, Beijing 100000, China; # Spallation Neutron Source Science Center, Dongguan 523000, China

**Keywords:** sodium-ion batteries, hard carbon, pore architecture, pore volume, pore filling

## Abstract

Hard carbon stands out as an auspicious anode material for commercial sodium-ion batteries, yet the correlation between plateau-potential capacity and its pore architecture remains poorly understood. In this study, we systematically investigated the sodium-ion storage behavior in hard carbons with tailored pore architecture. The plateau-potential capacity of hard carbon is attributed to the filling of sodium clusters within closed nanopores and open nanopores that are impervious to the solvent molecules of the electrolyte. Small-angle X-ray scattering (SAXS) has been shown to be an effective method for estimating the volume of nanopores that can store sodium clusters. A rapid and user-friendly butanol pycnometry technique is designed to assess the volume of nanopores available for sodium-ion storage. This method has established a linear correlation between the nanopore volume detected and the plateau-potential capacity measured experimentally. We identified two scenarios where the plateau-potential capacity deviates from the congruence linear relationship established by SAXS and butanol pycnometry techniques. First, sodium clusters are unable to fill nanopores larger than 4 nm and could only partially fill those larger than 2 nm. Second, the diffusion of Na^+^ ions is impeded in graphene nanodomains with tight interlayer spacing and extended crystalline planes.

## Graphical abstract

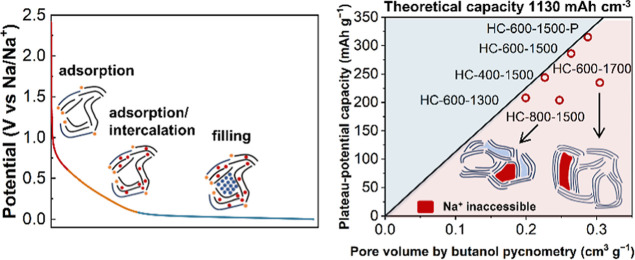

## 1 Introduction

The widespread employment of lithium-ion batteries (LIB) for large-scale energy storage faces challenges due to the limited availability and uneven distribution of lithium resources.^1,2^ In contrast, sodium resources in the ocean and the earth’s crust are more abundant than lithium resources. Therefore, low-cost sodium-ion batteries (SIBs) are promising alternatives to LIBs for large-scale energy storage applications.^3–5^ Hard carbon (HC) exhibits moderate sodium-ion storage capacities ranging from 200 to 300 mA h g^–1^ at a low potential plateau (around 0.1 V vs Na^+^/Na), outstanding rate capability, and long cycle life.^6–8^ With these attributes, HC emerges as the best choice for the anode material in commercial SIBs.^9^

HC is a family of nongraphitizable carbons that features closed pores (pores inaccessible to any substance without the force of an electric field due to the closure by graphene nanodomains (GNDs)) and open pores (probe molecules could penetrate) formed by the erratic arrangement of turbostratic GNDs.^10^ This unique amorphous–crystalline structure enables the capability for the storage of sodium ions in HC through adsorption in defects, intercalation in GNDs, and the pore filling of sodium clusters in “closed pores”, which results in a slope discharge curve with potentials higher than 0.1 V and a plateau discharge curve with potentials below 0.1 V.^11^

Scientists have demonstrated that the plateau-potential capacity (PPC) of HC originates from the filling of sodium clusters within nanopores.^9,12^ In 2000, Dahn observed decreased pore scattering intensity of small-angle X-ray scattering (SAXS) of HC discharged to the plateau-potential region. Dahn thus attributed the PPC to the filling of sodium ions in the nanopores.^13^ Yamada et al. observed the sodium (110) diffraction signals of wide-angle X-ray scattering (WAXS) in the plateau-potential region.^14^ Gray et al. identified the quasi-metallic characteristics of sodium clusters formed in the plateau-potential region through in situ solid-state nuclear magnetic resonance (ssNMR) spectroscopy.^15^ Komaba and Myung detected the signals of sodium clusters formed in the plateau-potential region through the in situ Raman technique.^16^ These findings indicate the PPC originates from the filling of nanopores by quasi-metallic sodium clusters.

Nevertheless, establishing a precise relationship between nanopore volume detected and PPC poses a challenge due to the difficulty in quantifying the quantity of filled sodium cluster and the complexity in characterizing the pore architecture. If all the space of nanopores are filled in by sodium clusters, a positive correlation could be expected between the nanopore volume detected and the actual PPC. However, due to the complexity of pore architecture, a clear relationship can be hardly established between PPC and pore structural parameters. In a previous work, the HC synthesized using MgO as a hard template had a true density of 1.99 g cm^–3^ (determined by helium pycnometry) and a rather low closed pore volume of 0.06 cm^3^ g^–1^ (in principle, pore volume calculated by helium pycnometry is regarded as closed pore volume), but still exhibited a remarkable PPC of 401 mA h g^–1^.^17^ This result indicates that the filling of sodium clusters is not limited to the closed pores. In contrast, HC with a true density of 1.392 g cm^–3^ and a corresponding closed pore volume of 0.277 g cm^–3^ should have a PPC of 313 mA h g^–1^, but the actual PPC tested was approximately 250 mA h g^–1^.^18^ The divergence between the theoretical PPC calculated from the pore volume detected and the actual PPC emerges consequently.

Hu et al. conducted theoretical calculations revealing that the density of sodium clusters in nanopores exceeds that of sodium metal.^19^ However, both ex-situ WAXS and in situ pair distribution function (PDF) analysis revealed that the lattice structure of sodium clusters closely resembles that of sodium metal.^14,15^ Yun et al. investigated the relationship between the volume fraction of nanopores (Φ_pore_ with respect to the volume of hard carbon) and the volume fraction of sodium filling (Φ_Na_ with respect to the volume of hard carbon) in HCs calculated from PPC.^20^ However, in this work, Φ_Na_ is calculated independently from the structural parameters of the nanopores in HC, and the PPC values can be hardly predicted using the structural parameters of hard carbons. Therefore, the establishment of an instructive relationship between PPC and the pore architecture in HC is of great significance, which could guide us in designing efficient HC anodes toward advanced SIBs.

The architecture of nanopores (pore shape, pore size, pore size distribution, parameters of GNDs that build the walls of pores, and pore aperture and pore body of open pores) in HCs determines the electrochemical sodium-ion storage behaviors of HCs.^21^ Yang et al. discovered that sodium clusters could be stored within pores inaccessible to solvent molecules of electrolyte, where the lack of accessibility of the solvent molecules enables the sodium ions to acquire electrons and form sodium clusters.^22^ However, for bottlenecked open pores (nanopores with small pore aperture sizes and large pore body sizes), the aperture sizes of the open pores that are inaccessible to the solvent molecules of the electrolyte are difficult to determine. Therefore, it is not easy to estimate the nanopore volume of bottle-necked open pores available for the formation of sodium clusters and to construct the precise relationship between PPC and nanopore architecture.

The GNDs with narrow interlayer spacing, excessive crystalline thickness, and elongated crystalline lengths would principally hinder the diffusion of sodium ions in HCs, leading to limited accessibility of sodium ions in nanopores and decreased pore-filling ratios.^23,24^ In one case, the metallicities of sodium clusters formed in nanopores with diameters of 3.56 and 2.41 nm are similar, indicating comparable sizes of the formed sodium clusters.^22^ This suggests that, in large nanopores, quasi-metallic sodium clusters are constrained in size, resulting in decreased pore-filling ratios. Density-functional theory (DFT) calculations show that the stability of sodium clusters decreases as the sodium cluster thickness increases.^25^ Therefore, deep insights into understanding how the nanopore architecture affects the formation of sodium clusters would facilitate the construction of a more precise relationship between PPC and nanopore volume tested.

In this contribution, we prepared HCs with tuned carbon architectures using sodium lignosulfonate (LS) as the carbon precursor by engineering the carbonization regime. We proposed the potential-dependent Na-ion storage mechanism in HC as “adsorption, adsorption/intercalation, and pore-filling”. The PPC originates from filling quasi-metallic sodium clusters into nanopores that are accessible to sodium ions but inaccessible to the solvent molecules of electrolytes. Generally, the helium pycnometry technique underestimates the nanopore volume due to the existence of some bottle-necked open nanopores. For the HCs with low preparation temperature (<1600 °C) and low specific surface areas, the nanopore volume for sodium cluster filling could be precisely estimated using SAXS. A butanol pycnometry technique that is fast and easy to operate was designed to evaluate the volume of nanopores for sodium ion storage. The pore-filling ratios calculated by dividing the sodium cluster volume by the nanopore volume tested and the actual pore-filling ratios determined from the nanopore density calculated using the ex-situ SAXS technique exhibit congruence. Based on our proposed butanol pycnometry method, a proportional relationship between nanopore volume detected and actual PPC was established. Discrepancies between the actual and theoretical PPCs stem from two main factors. First, sodium clusters could not fill nanopores larger than 4 nm and fill incompletely nanopores larger than 2 nm. Second, the diffusion of Na^+^ ions is interrupted in graphene nanodomains with small interlayer spacing and long crystalline planes, preventing Na^+^ ions from storing in nanopores with well-grown graphene nanodomains.

## 2 Results and Discussion

Through self-templated pore formation (blue arrow) and high-temperature heat treatment (red arrow) (Scheme 1), we manipulated the architecture of the closed and open pores of HC. The sodium sulfonate group of LS in situ formed inorganic self-templates at temperatures of 400 °C, 600 °C, and 800 °C. The obtained porous carbons with inorganic salts washed off were denoted as LSC-400, LSC-600, and LSC-800. The sodium sulfonate in LS displayed a diagonal crystalline Na_2_SO_4_ structure, which was transformed to a hexagonal phase at 400 °C (Figure 1a). The Na_2_SO_4_ in the sintered product of LSC400 only changed its crystallinity without any noticeable reaction with carbon. At the carbonization temperature of 600 °C, the sodium sulfonate group of LS reacted with carbon, resulting in the formation of the Na_4_CO_3_SO_4_ templates (Figure 1a). The N_2_ adsorption/desorption isotherms of LSC-600 showed typical H2-type hysteresis loops (Figure 1b), suggesting that the pores of LSC-600 displayed bottlenecked pore structures.^26^ The inorganic Na_4_CO_3_SO_4_ resulted in a self-templating effect, giving LSC-600 a specific surface area of 201.0 m^2^ g^–1^ (Figure 1b and Table S1). The micropore sizes of LSC-600 were predominantly focused at 1.5 nm (Figure 1c). At 800 °C, the template interacted with the carbon skeleton forming Na_2_CO_3_ (Figure 1a), causing the pore aperture to be enlarged and the inorganic template to precipitate out. This process results in the formation of wide-aperture open pores and the higher specific surface area (438.5 m^2^ g^–1^) of LSC-800 than that of LSC-600 (Figure 1b and Table S1). The pore size distribution of LSC-800 ranged from 0.5 to 0.9 nm and from 1 to 2 nm (Figure 1c).

**Scheme 1:**
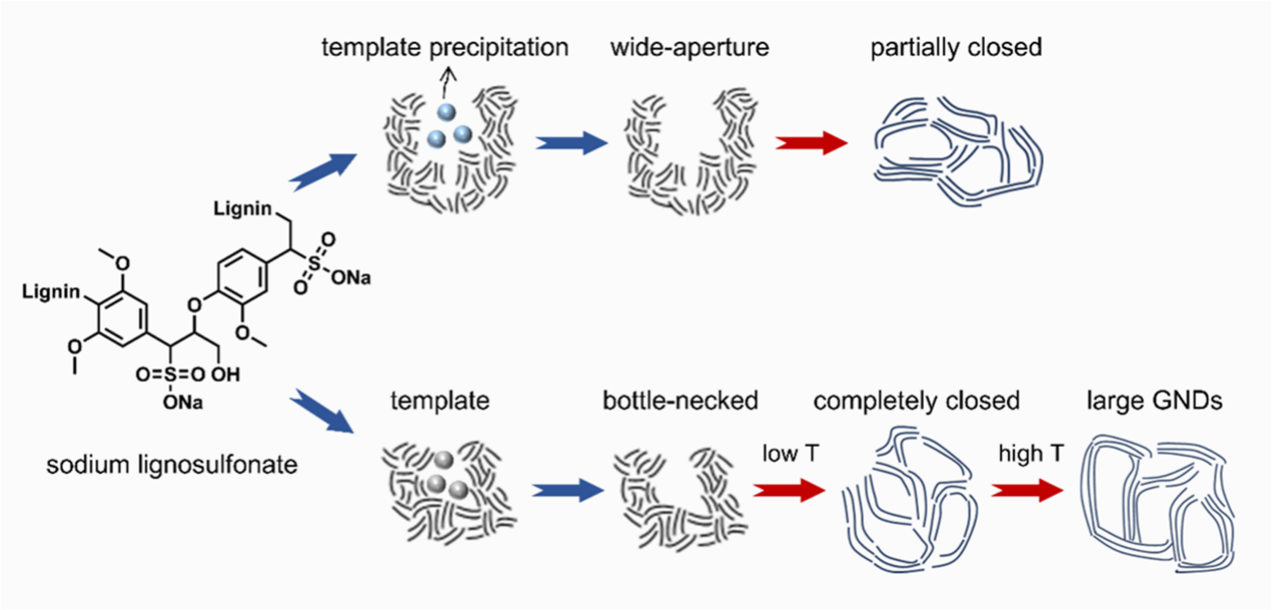
Schematic Illustrating the Evolution of the Pore Architecture in LS-Derived HCs

**Figure 1:**
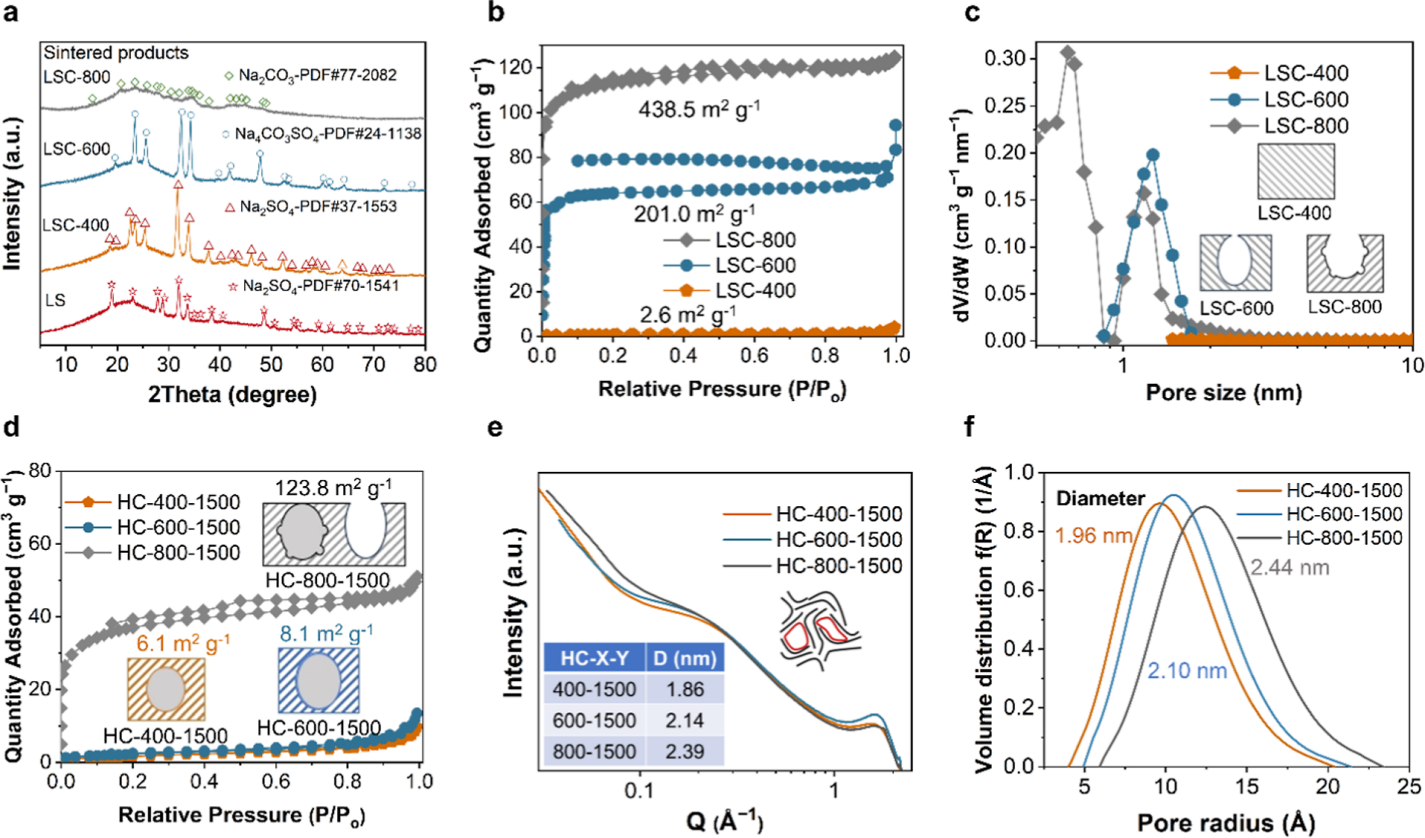
(a) XRD patterns for LS and sintered products of LS at 400, 600, and 800 °C. (b) N_2_ adsorption/desorption isotherms, and (c) pore size distribution for LSC-400, LSC-600, and LSC-800. The insets illustrate the lack of inner pore structure in the LSC-400, the existence of bottle-necked pore structure in the LSC-600, and wide-aperture open pore structure in the LSC-800, respectively. (d) N_2_ adsorption/desorption isotherms of HC-400–1500, HC-600–1500, and HC-800–1500. Gray and white colors signify that the pore is closed and open, respectively. The insets illustrate the closure of open pores, which shows that open pores are almost closed for HC-600–1500, partially closed for HC-800–1500, and completely closed for HC-400–1500. (e) SAXS patterns of HC-400–1500, HC-600–1500, and HC-800–1500. The insets show the model structure of HC and the pore diameter parameters (D), respectively. (f) Pore distribution curves were obtained from modeling the nanopore region of the SAXS data for HC-400–1500, HC-600–1500, and HC-800–1500.

Through high-temperature heat treatment of LSC samples, the open pores evolve into bottle-necked pores and eventually closed pores. The LSC-400, LSC-600, and LSC-800 samples underwent heat treatment at 1500 °C. The resulting HC samples are denoted as HC-400–1500, HC-600–1500, and HC-800–1500, respectively. The specific surface areas of HC-400–1500, HC-600–1500, and HC-800–1500, evaluated by N_2_ adsorption/desorption technique, were 6.1, 8.1, and 123.8 m^2^ g^–1^, respectively (Figure 1d and Table S2). It suggests that the bottle-necked pores in LSC-600 are more prone to closure, forming closed pores, compared to the wide-aperture pores in LSC-800. The CO_2_ adsorption/desorption isotherms of HC-400–1500, HC-600–1500, and HC-800–1500 all displayed H2-type hysteresis loops, indicating the presence of bottle-necked pore structures in these HC samples (Figure S2a,b). The specific surface areas of HC-400–1500, HC-600–1500, and HC-800–1500, determined via CO_2_ adsorption/desorption, were 40.5, 37.4, and 236.6 m^2^ g^–1^, respectively (Table S3). Furthermore, from the pore size distribution obtained from CO_2_ adsorption/desorption, HC-400–1500, HC-600–1500, and HC-800–1500 had ultramicropores (pore size smaller than 0.7 nm) (Figure S2c). Specifically, CO_2_ adsorption/desorption analysis revealed that HC-800–1500 contained open micropores below 0.85 nm (Figure S2c), while N_2_ absorption/desorption analysis showed that the open micropores in HC-800–1500 were predominantly in the range of 0.9 to 1.3 nm (Figure S3). This result indicates that the HC-800–1500 features some micropores that are inaccessible to N_2_ but accessible to CO_2_. The ultramicropore volume of HC-800–1500 was 0.041 cm^3^ g^–1^ (Table S3). As the solvent molecules of the electrolyte are larger than the N_2_ molecules (0.364 nm),^27^ these ultramicropores are inaccessible to the solvent molecules. Therefore, these opren ultramicropores have the potential to store sodium ions and host sodium clusters.

In contrast to LSC-600 and LSC-800, LSC-400 lacked open micropore structures (Figure 1b), while HC-400–1500 still had abundant nanopores, as indicated by the SAXS curves (Figure 1e). The nanopores in HC-400–1500 were formed through the elimination of heteroatoms and structural rearrangement of the carbon skeleton during the heat treatment process (Tables S4 and S5). Furthermore,the average nanopore diameters for HC-400–1500, HC-600–1500, and HC-800–1500 were simulated as 1.86, 2.14, and 2.39 nm by fitting the SAXS curves using the Teubner–Strey model (Figure 1e and Table S6). The nanopore volume fractions of HC-400–1500, HC-600–1500, and HC-800–1500 were 33.5%, 37.2%, and 37.5%, respectively (Table S6). The HCs prepared through a self-templating strategy would achieve a larger pore volume compared to HCs prepared without employing templates. The interlayer spacing (d_002_) of HC-400–1500, HC-600–1500, and HC-800–1500 were calculated as 0.380, 0.381, and 0.371 nm, and the number of graphene layer stacks (N) were 4.03, 4.05, and 4.80, respectively (Figure S5 and Table S8). The GNDs of HC-800–1500 shrank more densely than those of HC-400–1500 and HC-600–1500, resulting in a larger nanopore diameter in HC-800–1500 compared to HC-600–1500 and HC-400–1500.

In addition, the pore size distribution of the nanopores could be fitted by the Schulz-Zimm pore distribution model (Note S4 in Supporting Information). The average pore diameters of HC-400–1500, HC-600–1500, and HC-800–1500 were fitted as 1.96, 2.10, and 2.44 nm, respectively (Figure 1f and Table S7). The full width at half-maximum (fwhm) values of the pore size distributions for HC-600–1500 and HC-800–1500 were 6.34 and 6.20 Å, respectively (Table S7). Since LSC-800 has a broader pore size distribution compared to LSC-600, the nanopore size distribution of HC-800–1500 was wider than that of HC-600–1500. The LSC-400 lacked open pore structures, thus HC-400–1500 exhibited a smaller fwhm of 5.81 Å in contrast to HC-600–1500 and HC-800–1500.

If HC is subjected to higher heat treatment temperatures, the GNDs in HC become well-grown as the interlayer spacing shrinks and the crystalline size increases, resulting in larger closed pore size and volume.^28,29^ The LSC-600 underwent heat treatment at 1300 and 1700 °C to adjust the GNDs and pore structures of HC. The obtained HCs were denoted HC-600–1300 and HC-600–1700. Additionally, a sample denoted as HC-600–1500 P was prepared by implementing a soft carbon surface coating layer using pitch as the precursor. This was done to mitigate the formation of open pores and defects in HCs and to investigate the influence of soft carbon coating on the regulation of pore architecture. As the heat treatment temperature increased, the average *d*_002_ of HC-600–1300, HC-600–1500, and HC-600–1700 decreased from 0.391 nm gradually to 0.368 nm (Figure 2a). The local narrow *d*_002_ of HC-600–1700 could impede the diffusion of Na^+^ ions, which deteriotes the specific capacity.^30^ Simultaneously, the length of the crystalline plane (*L*_a_) increased from 4.39 to 6.51 nm, the thickness of the crystalline plane (*L*_c_) increased from 0.86 to 1.95 nm, and the graphene stacking number *N* increased from 3.23 to 6.34 (Figure 2a and Table S8). As the heat treatment temperature increased, the graphene layer of HC became denser. The *d*_002_, *L*_a_, and *L*_c_ values of HC-600–1500 P were 0.383, 4.82, and 1.18 nm, respectively, showing a slight increase compared to HC-600–1500 due to surface coating by soft carbon (Figure 2a). HC-600–1500 P should have a comparable inner crystalline microstructure compared with HC-600–1500. The soft carbon coating on the surface does not affect the main crystalline structure of HCs under the same carbonization temperature, but the pore architecture may be altered.

**Figure 2:**
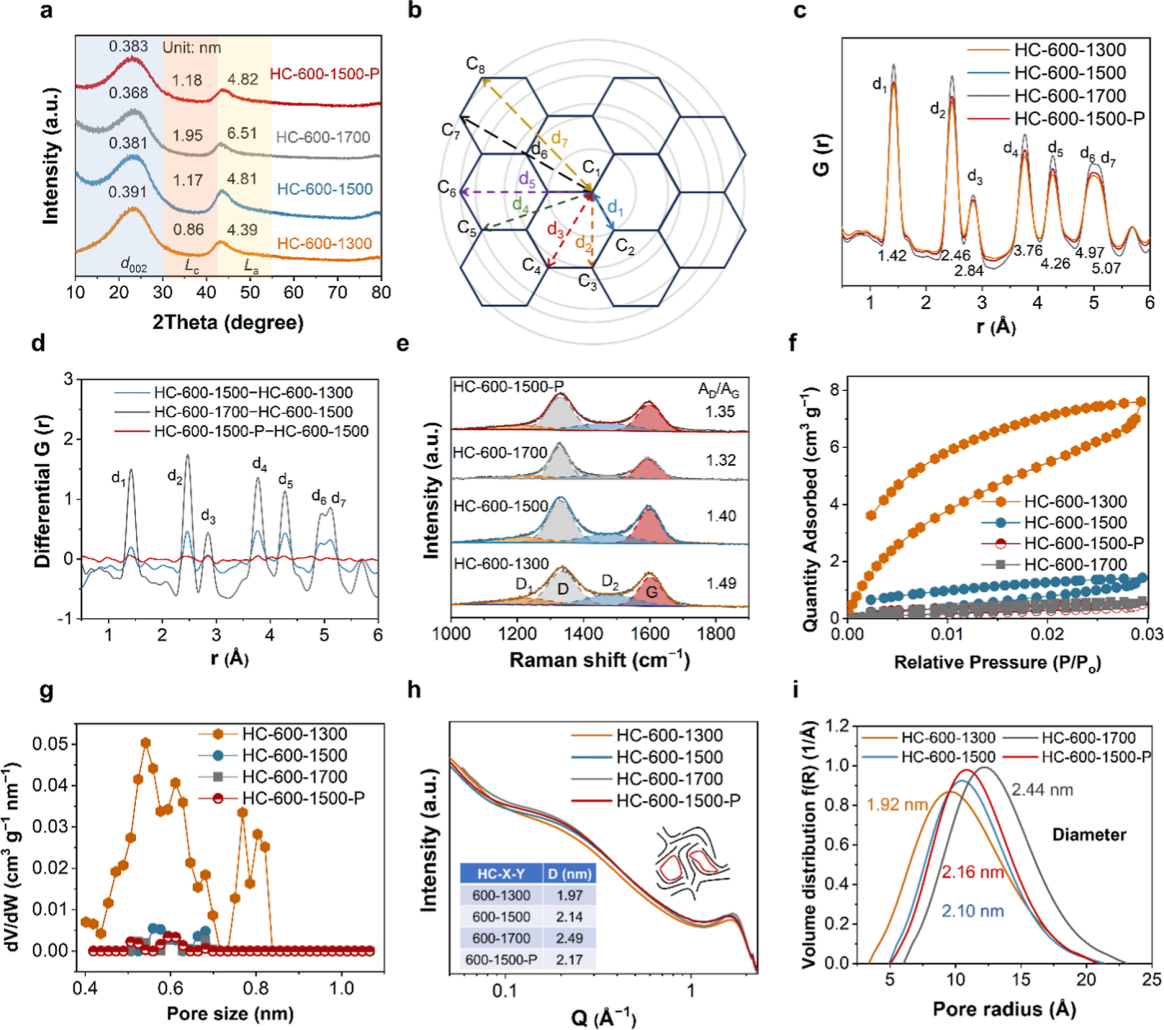
(a) XRD patterns, (b) schematic of the carbon interatomic distances, (c) PDF analysis obtained from neutron total scattering, (d) differential PDF curve between HCs, (e) Raman spectroscopy, (f) CO_2_ adsorption/desorption isotherms, (g) pore size distribution obtained from CO_2_ adsorption/desorption, and (h) SAXS patterns for HC-600–1300, HC-600–1500, HC-600–1700, and HC-600–1500 P. The insets show the structural model of HC and the pore architecture, respectively. (i) Pore size distribution curve obtained from modeling the nanopore region of the SAXS data for HC-600–1300, HC-600–1500, HC-600–1700, and HC-600–1500 P. The data shows the average pore sizes.

The PDF analysis obtained from neutron total scattering was employed to characterize the carbon–carbon distances (*r*) in HC (Figures 2b and S5b). The *G*(*r*) values of peaks ranging from d_1_ to d_7_ exhibited an increase with rising heat treatment temperatures (Figure 2c). This trend indicates that as the temperature rises, the graphene layer thickens and the graphene size grows. The difference in the G­(*r*) value (Δ*G*(*r*)) between the HC-600–1700 and HC-600–1500 exceeded the Δ*G*(*r*) value between HC-600–1500 and HC-600–1300 (Figure 2d). It indicates that the growth of crystalline planes in HC from temperatures ranging from 1500 to 1700 °C was more extensive compared to the growth from temperatures ranging from 1300 to 1500 °C, which could lead to an apparent influence on the pore architecture of HC. Compared to HC-600–1500, HC-600–1500 P only displayed a slight increase in *G*(*r*) signal at the d_1_, d_2_, and d_3_ peaks (Figure 2d), attributable to the small portion of well-grown graphene layers on the surface of HC-600–1500 P.

As the heat treatment temperature increases, the growth of the graphene layer of HC leads to a decrease in defects. The *A*_D_/*A*_G_, *A*_D1_/*A*_G_, and *A*_D2_/*A*_G_ values indicate the degree of intrinsic defects and edge defects and the sp^3^ carbon content in HC, respectively (the detailed calculation method is shown in Note S1 of Supporting Information).^31,32^ The *A*_D_/*A*_G_ values for HC-600–1300, HC-600–1500, and HC-600–1700 decreased from 1.49 to 1.32, the value of *A*_D1_/*A*_G_ decreased from 0.49 to 0.30, and *A*_D2_/*A*_G_ decreased from 0.85 to 0.47 (Figure 2e and Table S9). The increase in the heat treatment temperature was found to result in a decrease in intrinsic defects, edge defects, and the content of sp^3^ hybridized carbon atoms linking the local GNDs in HCs.

The growth of the graphene layer is accompanied by variations in the open pores of HCs. The CO_2_ adsorption/desorption isotherms of HC-600–1300, HC-600–1500, HC-600–1700, and HC-600–1500 P exhibited H2-type hysteresis loops (Figure 2f). The pore size distribution analysis showed that HC-600–1300, HC-600–1500, HC-600–1700, and HC-600–1500 P contained open micropores with sizes below 0.85 nm. And based on the pore size distribution curves obtained from N_2_ adsorption/desorption, HC-600–1300, HC-600–1500, HC-600–1700, and HC-600–1500 P lacked open micropores with diameters <1 nm (Figure S6b). These indicate that these HCs contained open bottle-necked pores inaccessible to N_2_ but accessible to CO_2_. The specific surface areas of HC-600–1300, HC-600–1500, HC-600–1700, and HC-600–1500 P, as determined by CO_2_ adsorption/desorption, were 62.9, 37.4, 18.3, and 14.4 m^2^ g^–1^, respectively (Table S3). The open bottle-necked pores content of HC decreased with higher heat treatment temperatures (Figure 2f and Table S3). Owing to the larger d_002_ and shorter *L*_a_ of HC-600–1300 than HC-600–1500, HC-600–1700, and HC-600–1500 P, it is feasible for CO_2_ to enter the bottle-necked pores of HC-600–1300 through nanopore apertures,^33^ which enables that HC-600–1300 exhibits a higher proportion of open ultramicropores (<0.7 nm) compared with the other HCs (Figure 2g).

The nanopore diameters of the HC increased as the graphene layer densified at higher temperatures. The nanopore diameters of HC-600–1300, HC-600–1500, HC-600–1700, and HC-600–1500 P were calculated as 1.97, 2.14, 2.49, and 2.17 nm by fitting the SAXS curves using the Teubner–Strey model (Figure 2h). The degree of connectivity of the nanopores in HC could be characterized by the “amphiphilicity factor” (*f*_a_).^34^ A higher *f*_a_ value indicates higher deviation from a spherical shape of the nanopores in HC, as well as increased pore connectivity. The *f*_a_ values for HC-600–1300, HC-600–1500, and HC-600–1700 were 0.50, 0.64, and 0.74, respectively (Table S6). With increasing carbonization temperature, the nanopores in HC undergo a merging process, transitioning into elliptical shapes and resulting in the overall enlargement of pore diameter. The pore merging decreases the overall number of nanopores, as evident in the pore number counts per 1000 nm^3^ for HC-600–1300, HC-600–1500, and HC-600–1700, which were 75.7, 72.5, and 51.3, respectively (Table S6). This results in a narrower nanopore size distribution with increasing heat treatment temperature. The fwhm of the pore size distributions of HC-600–1300, HC-600–1500, and HC-600–1700 were 6.69, 6.20, and 5.86 Å (Figure 2i and Table S7). The enlarged pore diameter and narrowed pore distribution are attributed to the growth of the graphene layer and the merging of pores.

The evolution of the nanopore architecture of HC tuned by self-templating technique is influenced by heat treatment temperature (Scheme 2). Although LSC-400 initially lacks open pore structure, pyrolytic removal of heteroatoms and the rearrangement of GNDs during the heat treatment process facilitate the development of nanopores in HC-400–1500. The bottle-necked nanopores in LSC-600 exhibited a higher propensity to close compared to the wide-aperture pores in LSC-800 during the heat treatment process. The incomplete closure of the wide-aperture pores in LSC-800 led to the presence of some N_2_-accessible micropores in HC-800–1500. HC-400–1500, HC-600–1500, and HC-800–1500 all contained bottle-necked pores accessible to CO_2_ but not accessible to N_2_. Differently, HC-800–1500 contained more of these bottle-necked pores. These bottle-necked pores have the potential to store sodium clusters as they do not allow solvent molecules to fill in.

**Scheme 2:**
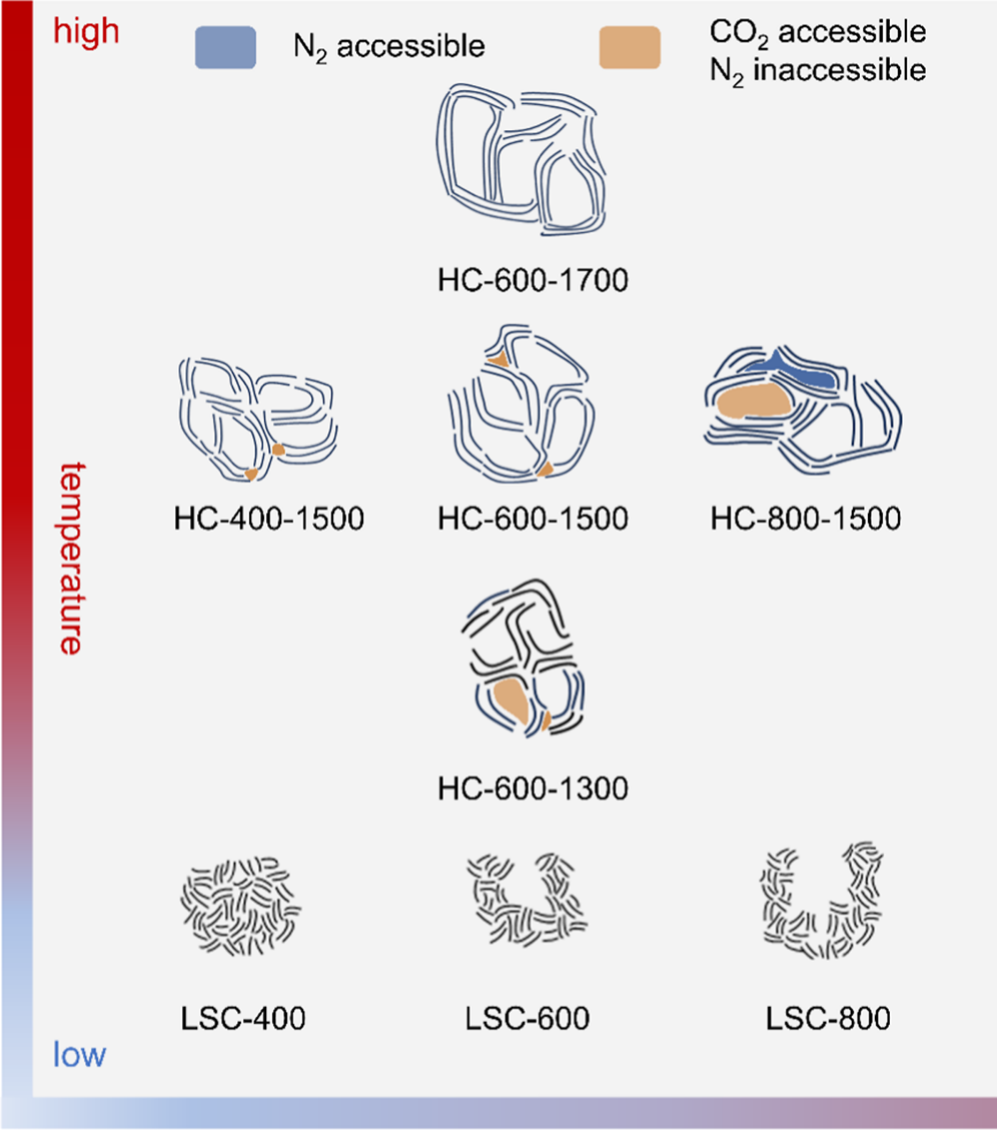
Schematic of the Evolution of Pore Architecture in HC with Increasing Temperature

As the temperature increased, the *d*_002_ of HC became narrower, *L*_a_ and *L*_c_ elongated, the number of graphene layer stacks increased, the defect concentration decreased, and the number of open pores decreased. Due to the large *d*_002_ and short *L*_a_, HC-600–1300 possessed abundant bottle-necked pores that were inaccessible to N_2_ but accessible to CO_2_. Furthermore, raising the heat treatment temperature caused the merging of nanopores in HC, enlarging the diameters of nanopores, diminishing the number of pores, and narrowing the pore size distribution.

The charge/discharge curves of the HC exhibited distinct plateau-potential and slope-potential regions (Figures 3a and S8). In the discharge process, the plateau-potential region was identified as the range where the derivative of specific capacity to potential (dQ/dV) was lower than −400 mA h g^–1^ V^–1^, while the dQ/dV values higher than −400 mA h g^–1^ V^–1^ corresponded to the slope-potential region (Figure S9). Specifically, HC-600–1300, HC-600–1500, HC-600–1700, HC-600–1500 P, HC-400–1500, and HC-800–1500 displayed slope-potential capacities of 106, 98, 74, 99, 92, and 100 mA h g^–1^, respectively, at a current density of 20 mA g^–1^ (Figure 3b). Notably, at the same heat treatment temperature, the HC-400–1500, HC-600–1500, HC-600–1500 P, and HC-800–1500 samples exhibited similar slope-potential capacities. While the slope-potential capacities of HC-600–1300, HC-600–1500, and HC-600–1700 decreased with increasing the temperature of heat treatment. The PPCs of HC-600–1300, HC-600–1500, HC-600–1700, HC-600–1500 P, HC-400–1500, and HC-800–1500 were 208, 286, 235, 315, 244, and 204 mA h g^–1^ (Figure 3b). The nanopore volume of HC-600–1700 (0.307 cm^3^ g^–1^) was larger than that of HC-600–1500 (0.262 cm^3^ g^–1^) (Table S6). If the PPC is solely attributed to sodium cluster filling in nanopores, the PPC of HC-600–1700 should exceed that of HC-600–1500. However, the actual PPC of HC-600–1700 (235 mA h g^–1^) was much lower than that of HC-600–1500 (286 mA h g^–1^). It is possible that the distinct pore architectures of HC influence the PPC.

**Figure 3:**
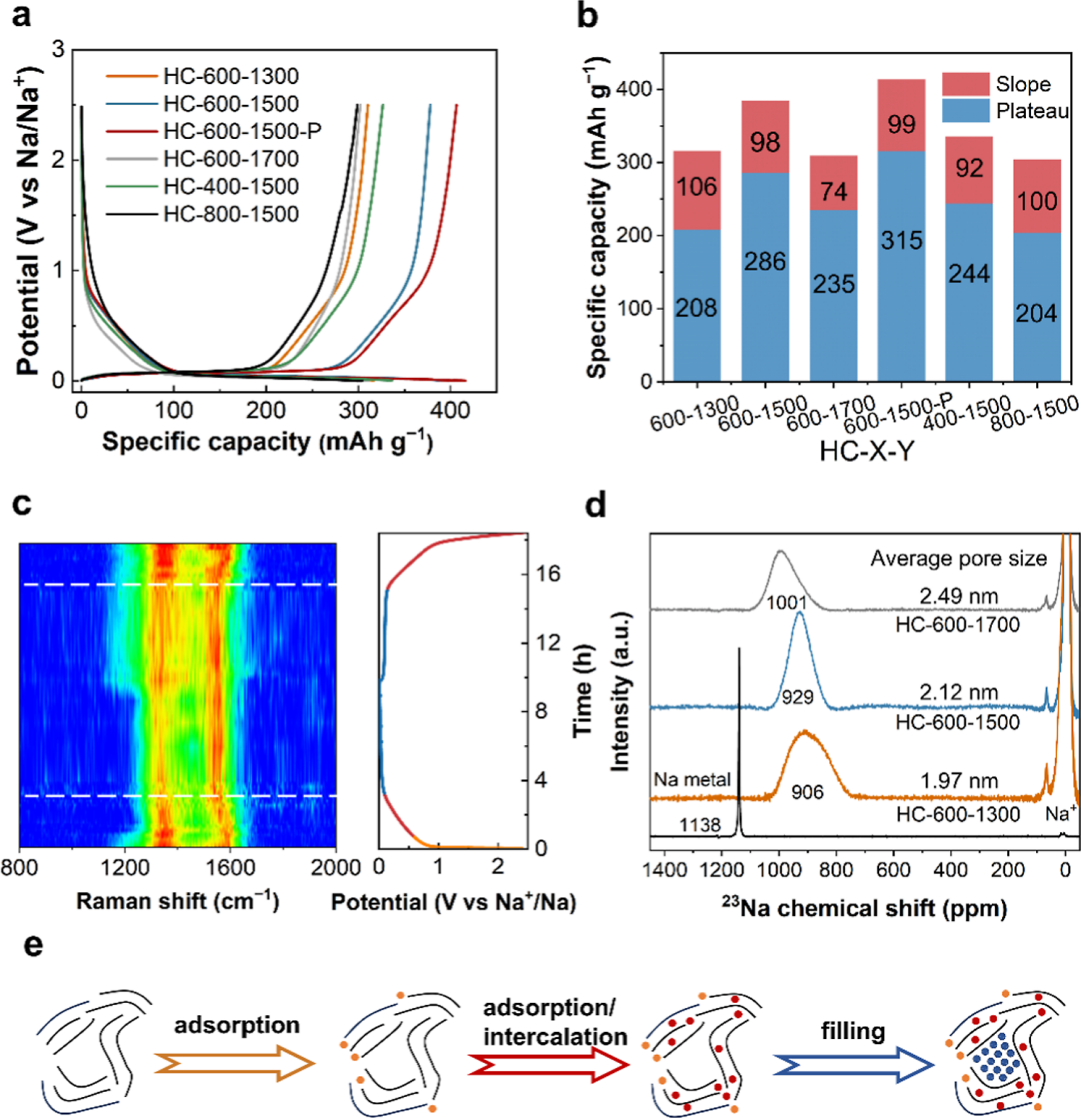
(a) Galvanostatic charge/discharge curves in the second cycle at a current density of 20 mA g^–1^, and (b) statistics of slope-potential capacity and PPC for HC-600–1300, HC-600–1500, HC-600–1500 P, HC-600–1700, HC-400–1500, and HC-800–1500 samples. (c) In situ Raman spectrum of HC-600–1300 during discharge/charge processes at 20 mA g^–1^. (d) Ex situ ^23^Na MAS ssNMR of HC-600–1300, HC-600–1500, and HC-600–1700 sodiated to 0.001 V at a current density of 20 mA g^–1^. (e) Schematic illustrating the sodium-ion storage mechanism in HCs.

The sodium-ion storage mechanisms of HC were investigated using in situ Raman spectroscopy, ex-situ XRD, ex-situ WAXS, ex-situ SAXS, and ex-situ ^23^Na MAS ssNMR. During the discharge from 0.9 to 0.6 V, the in situ Raman analysis revealed weakening of the D band intensity, and constant of the G band intensity for HC-600–1300. In the discharge process from 0.6 to 0.1 V, the intensity of the D band decreased, accompanied by a shift in the D and G bands toward lower Raman shifts (Figure 3c). At the onset of discharge, the transfer of electrons to the defects on the surface of HC results in the interaction between Na^+^ ions and defects, which in turn induces a decrease in the intensity of the D band.^35,36^ Subsequently, Na^+^ ions intercalate into the GNDs, reaching the internal defect sites. During the intercalation of Na^+^ ions into the GNDs, electrons occupy the carbon–carbon π*-antibonding orbitals of GNDs and interact with Na^+^ ions, leading to an increase in the C–C bond length, accompanied by a subsequent shift in the G band.^15,16,35–37^ Thus, the mechanism of sodium-ion storage in the slope-potential region is adsorption and adsorption/intercalation. Na^+^ ions are difficult to intercalate in GNDs with *d*_002_ smaller than 0.36 nm.^38^ The increase in heat treatment temperature resulted in a decrease in defects and the contraction of interlayer spacing in HC (Tables S8 and S9), consequently diminishing the slope-potential capacity. In the plateau-potential region, the intensity and Raman shift of the G and D bands did not change (Figure 3c), indicating the absence of intercalation and adsorption processes.

The d_002_ value of HC-600–1300 at a sodiation potential of 0.1 V expanded to 0.397 nm, slightly exceeding that of the d_002_ (0.390 nm) of the pristine electrode (Figure S10). This slight increase was attributed to the Na^+^ intercalation within the GNDs. The d_002_ of the HC-600–1300 at sodiation potentials from 0.1 to 0.001 V was unchanged (Figure S10). The intercalation process of Na^+^ ions did not definitely lead to a change in the d_002_. The HC prepared by Li et al. had a large d_002_ of 0.409 nm, and the d_002_ of this HC did not change during the sodiation process.^19^ A large d_002_ potentially has a large enough space to accommodate the intercalation of Na^+^ ions without a change in the d_002_. While the HC (CNF-2200) prepared by Zhang et al. had a small d_002_ of 0.348 nm.^39^ The d_002_ of CNF-2200 also remained unchanged during the sodiation process. CNF-2200 has a low slope-potential capacity of about 40 mA h g^–1^. The reason for the unchanged d_002_ of CNF-2200 during sodiation process could be that the slope-potential capacity of CNF-2200 was very low and mainly contributed by adsorption of Na^+^ ions, with little contribution from intercalation of Na^+^ ions. Compared to the d_002_ variation in the XRD patterns, the position and intensity of the G-band in the Raman spectrum enable easier determination of the intercalation of Na^+^ ions.

Additionally, ex-situ XRD revealed the presence of diffraction peaks (2theta = 29.45°) from sodium clusters in HC-600–1300 sodiated to 0.04 V (Figure S10). The intensity of the sodium cluster diffraction peak increased notably for HC-600–1300 sodiated to 0.001 V (Figure S10), which is ascribed to the progressive filling of sodium clusters. Through ex-situ WAXS analysis, the scattering peak of quasi-metallic sodium clusters at *Q* = 2.072 Å^–1^, corresponding to a diffraction angle of 2-theta = 29.45°, consistent with the (110) crystalline plane of metallic sodium (Figure S11). This observation aligns with the findings of Yamada et al., who previously detected the diffraction peak at a *Q* value of 2.072 Å^–1^ for the sodium clusters using WAXS.^14^ The Na-to-Na distance of sodium clusters, determined through DFT-MD, was found to be 3.7 Å, equivalent to that of bulk sodium metal.^6,40,41^ Gray and her colleagues determined that the PDF signals in the plateau-potential region originated from the sodium microcrystals with a size ranging from 5 to 20 Å, through PDF differential analysis.^15^ Our results and the previous results suggest that the lattice structure of quasi-metallic sodium clusters closely resembled that of metallic sodium, indicating the similar density between sodium clusters and bulk sodium.

The pore scattering intensity of the HC electrode sodiated to 0 V was weaker than that of the pristine electrode, as evidenced by ex-situ SAXS analysis (Figures S12 and S13). This decrease in pore scattering intensity was ascribed to the decrease in density contrast between the carbon matrix and the pores due to the filling of sodium clusters.^42,43^ The ^23^Na chemical shifts of sodium clusters within the nanopores of HC-600–1300, HC-600–1500, and HC-600–1700 were 906, 929, and 1001 ppm, respectively (Figure 3d). The ex-situ ^23^Na ssNMR spectroscopy revealed that the sodium clusters occupying the nanopores of HC exhibited quasi-metallic characteristics (Figure 3d).^44^ The chemical shift of the quasi-metallic sodium clusters formed in nanopores of HC increases with the increase of the pore diameters, approaching that of metallic sodium (Figure 3d). This suggests that nanopores with larger diameters allow lower valence states of sodium clusters to form, which results in lower potential for the formation of sodium clusters (Figure S9).

In short, it has been established that the slope-potential capacity of HC is a result of Na^+^ adsorption on defects and intercalation within GNDs (Figure 3e). During the discharge from 0.9 to 0.6 V, Na^+^ ions adsorbed onto surface defects. During the discharge from 0.6 to 0.1 V, Na^+^ ions intercalate into the GNDs, reaching the internal defect sites. The increase in heat treatment temperature resulted in a decrease in defects and the contraction of interlayer spacing in HC, consequently diminishing the slope-potential capacity. While the PPC of HC originates from the filling of quasi-metallic sodium clusters in the nanopores (Figure 3e). The sodium-ion storage mechanism in the plateau-potential region should be intricately associated with the pore architecture. In the following section, we discuss the relationship between sodium-ion storage behavior in the plateau-potential region and the pore architecture.

SAXS has proven to be a sensitive technique for detecting scattering signals in samples with density variations.^45^ The decline in pore scattering intensity (*I*_pore_) after full sodiation is related to the increased pore density after the filling of sodium clusters (Figure 4a and S13). The density of the pores with sodium clusters filled in was estimated by analyzing the relationship between *I*_pore_ and ΔSLD^2^ (the calculation process is shown in Note S5 of the Supporting Information). The pore densities after sodium cluster filling for HC-600–1300, HC-600–1500, HC-600–1700, HC-600–1500 P, HC-400–1500, and HC-800–1500 were 0.875, 0.891, 0.644, 0.898, 0.872, and 0.663 g cm^–3^, respectively (Figure 4b and Table S10). The pore-filling ratios for HC-600–1300, HC-600–1500, HC-600–1700, HC-600–1500 P, HC-400–1500, and HC-800–1500 were calculated as 90.4%, 92.0%, 66.5%, 92.7%, 90.1%, and 68.5%, respectively (Table S10). HC-600–1700 and HC-800–1500 exhibited significantly lower pore-filling ratios compared to the other HCs. Although HC-600–1700 and HC-800–1500 have higher nanopore volumes than HC-600–1500, their pore architecture was not conducive to the efficient filling of sodium clusters.

**Figure 4:**
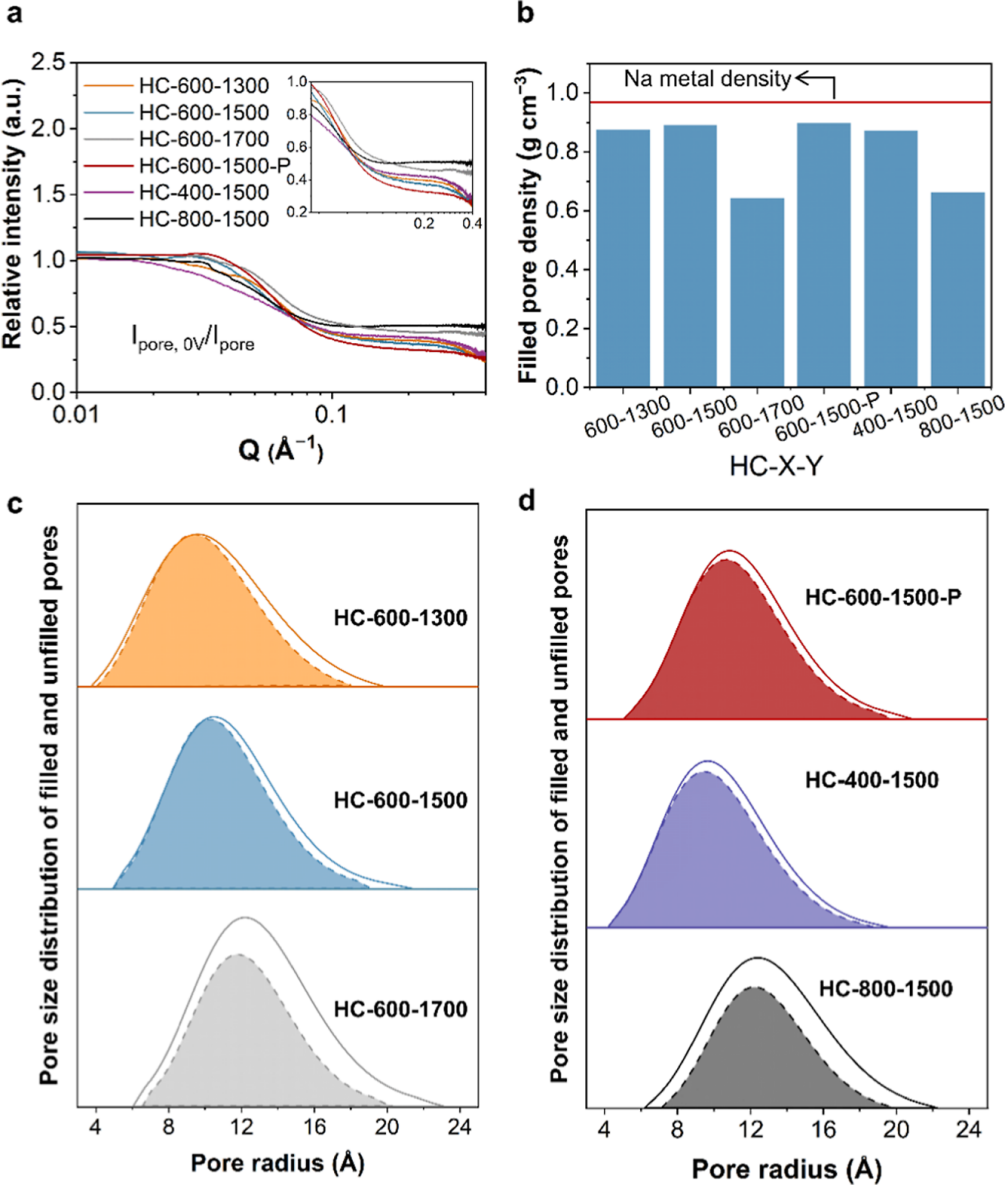
(a) Relative SAXS profiles of fully sodiated HC samples versus pristine electrodes. (b) Density of the pores after being filled for HC-600–1300, HC-600–1500, HC-600–1700, HC-600–1500 P, HC-400–1500, and HC-800–1500. Normalized pore size distribution of pristine HC and HC samples at a sodiation potential of 0.001 V for (c) HC-600–1300, HC-600–1500, HC-600–1700, (d) HC-600–1500 P, HC-400–1500, and HC-800–1500.

Furthermore, the pore size distribution of the HC was analyzed by SAXS both before and after the filling of sodium clusters (the calculation method is shown in Note S5 in the Supporting Information). The SAXS relative intensity curves illustrated a uniform decrease in scattering intensity across nanopores in the *Q* range from 0.08 to 0.4 Å^–1^ (Figure 4a), indicating that the pores were uniformly filled.^46^ Analysis of the nanopore size distributions in HCs before and after the filling of sodium clusters revealed a decrease in the pore-filling ratio of nanopores with diameters exceeding 20 Å (Figure 4c,d). Yong et al. previously demonstrated that the formation of larger sodium clusters requires a higher energy barrier and large sodium clusters (>2.17 nm) are unstable.^25^ Our experimental results align well with Yong’ results. For HC-600–1700 and HC-800–1500, the formation of sodium clusters within pores larger than 40 Å was notably limited. The larger sodium clusters form with lower formation potentials (higher formation energy barrier).^25^ For nanopores larger than 40 Å, the formation potential of sodium clusters is below 0 V vs Na^+^/Na (Figure S14), which indicates the inability of sodium clusters to fill in the pores larger than 40 Å in HC-800–1500 and HC-600–1700.

HC-600–1700 and HC-800–1500 displayed smaller d_002_ and larger *L*_a_ and *L*_c_ compared to other HCs (Table S8), indicating a denser graphene layer structure in the local GND. This dense structure hinders the sodium ion diffusion into the pores built by the walls of GND, preventing the formation of sodium clusters.^30^ The charge transfer resistances (*R*_ct_) of HC-600–1700 and HC-800–1500 were 139.3 Ω and 126.3 Ω, respectively. In contrast, HC-600–1300, HC-600–1500, HC-600–1500 P, and HC-400–1500 showed *R*_ct_ values of 45.6 Ω, 46.2 Ω, 48.4 Ω, and 40.6 Ω, respectively (Figure S15 and Table S13). The higher *R*_ct_ value indicates increased resistance to acquire charge during the sodiation process, which could result from the high energy barrier for sodium cluster formation and sluggish diffusion of sodium ions within GNDs.^24^ Furthermore, the *b* value obtained from cyclic voltammetry (CV) analysis characterizes the sodium-ion storage kinetics of HC for the process of filling sodium clusters (Figures S16 and S17). A *b* value of 0.5 signifies a diffusion-controlled process with slow electrode kinetics, while a *b* value of 1 indicates a near-surface-controlled process with rapid kinetics.^32^ The *b* values for HC-600–1300, HC-600–1500, HC-600–1700, HC-600–1500 P, HC-400–1500, and HC-800–1500 were 0.52, 0.51, 0.30, 0.50, 0.60, and 0.34, respectively (Figure S17). HC-600–1700 and HC-800–1500 demonstrated slower kinetics compared to the other HCs. The higher *R*_ct_ and the lower *b* values of HC-600–1700 and HC-800–1500 are attributed to the large energy barrier for the formation of sodium clusters in oversized nanopores and the slow diffusion of sodium ions in the dense GNDs. Consequently, sodium clusters were unable to fill some small nanopores with diameters smaller than 20 Å for HC-800–1500 and HC-600–1700, due to the dense GNDs restricting the access of Na^+^ ions (Figure 4c,d).

Therefore, for ideal HCs, the closed pores should be abundant, and small, while the GNDs are not well-grown. Moreover, the low pore-filling ratio of HC-800–1500 was ascribed to the presence of open pores accessible to the solvent molecules of the electrolyte. HC-800–1500 featured open pore volumes of approximately 0.014 and 0.016 cm^3^ g^–1^ for pore sizes ranging from 0.9 to 1.3 nm and 1.8 to 3 nm, as determined by N_2_ adsorption/desorption (Figure S3b and Table S2). The subsequent section delves into the impact of HC-800–1500s open pore volume on its pore-filling behavior of sodium.

To establish the correlation between PPC and pore architecture (pore shape, pore size, and pore accessibility), we assessed the pore volume of HC using the conventional helium pycnometry, SAXS, and our proposed butanol pycnometry. The true densities measured by helium pycnometry for HC-600–1500 and HC-800–1500 were tested to be 2.120 and 1.996 g cm^–1^, respectively, close to the density of graphite (2.258 g cm^–1^) (Table S14). The closed pore volume of HC can be calculated from the true density by eq 1

1 $$V_{closed\;pore}=\frac{1}{\rho_{true}}-\frac{1}{\rho_{struc}}$$

where *V*_closed pore_ is the closed pore volume of HC, ρ_true_ is the true density of HC, and ρ_struc_ is the structure density of HC. The closed pore volumes of HC-600–1300 and HC-800–1500 were thus calculated as 0.021 and 0.070 cm^3^ g^–1^ (Table 1 and S14), theoretically offering PPC of 23.7 and 79.1 mA h g^–1^. The theoretical PPC is calculated using eq 2

2 $$Q_{T,plat}=V_{closed\;pore}\times \rho_{Na}\times Q_{Na}$$

where *Q*_T,plat_ is the theoretical PPC of HC, ρ_Na_ is the density of sodium clusters (0.968 g cm^–3^), and *Q*_Na_ is the theoretical gravimetric specific capacity of sodium (1166 mA h g^–1^). The pore-filling ratio was calculated by the ratio of the volume demanded for the formation of sodium clusters (*V*_demand_) calculated from the actual PPCs to the pore volume tested. The *V*_demand_ was calculated using eq 3

3 $$V_{demand}=\frac{Q_{plat}}{\rho_{Na}\times Q_{Na}}$$

where *Q*_plat_ is the actual PPC of HC. The HC-600–1300 and HC-800–1500 require at least pore volumes of 0.184 and 0.181 cm^3^ g^–1^ (Table 1), respectively, to offer plateau capacities of 208 and 204 mA h g^–1^ (Table 1). Therefore, the closed pore volumes of HC-600–1300 and HC-800–1500, assessed by helium pycnometry, were significantly lower than the pore volumes required for the formation of sodium clusters (Figure 5a and Table 1). This implies that sodium clusters could be filled in the available open nanopores, in which the solvent molecules of electrolytes are inaccessible.

**Table 1:** Pore Volumes and Pore-Filling Ratios Obtained by Helium Pycnometry, SAXS, and Butanol Pycnometry

sample	closed pore volume by He pycnometry (cm^3^ g^–1^)	pore volume by SAXS (cm^3^ g^–1^)	pore volume by butanol pycnometry (cm^3^ g^–1^)	volume of sodium cluster^a^ (cm^3^ g^–1^)	filling ratio by closed pore volume (He pycnometry)^b^	filling ratio by pore volume (SAXS)^b^	filling ratio by pore volume (butanol pycnometry)^b^
HC-600–1300	0.021	0.196	0.199	0.184	876.2%	93.9%	92.3%
HC-600–1500	0.244	0.262	0.264	0.253	103.3%	96.5%	96.0%
HC-600–1700	0.302	0.307	0.304	0.208	68.9%	67.7%	68.3%
HC-600–1500 P	0.271	0.286	0.287	0.278	102.3%	97.0%	96.9%
HC-400–1500	0.171	0.224	0.226	0.216	126.3%	96.4%	95.6%
HC-800–1500	0.070	0.259	0.237	0.181	258.6%	69.8%	76.4%

a The volume of sodium clusters was calculated based on the actual PPC. The volume of sodium clusters was calculated using the formula $V_{demand}=\frac{Q_{plat}}{\rho_{Na}\times Q_{Na,m}}$ , where *Q*_plat_ is the PPC, ρ_Na_ is the density of sodium cluster (0.968 g cm^–3^), and *Q*_Na,m_ is the theoretical gravimetric specific capacity of sodium (1166 mA h g^–1^).

b The pore-filling ratio was calculated from the ratio of the volume of sodium clusters to pore volume tested.

**Figure 5:**
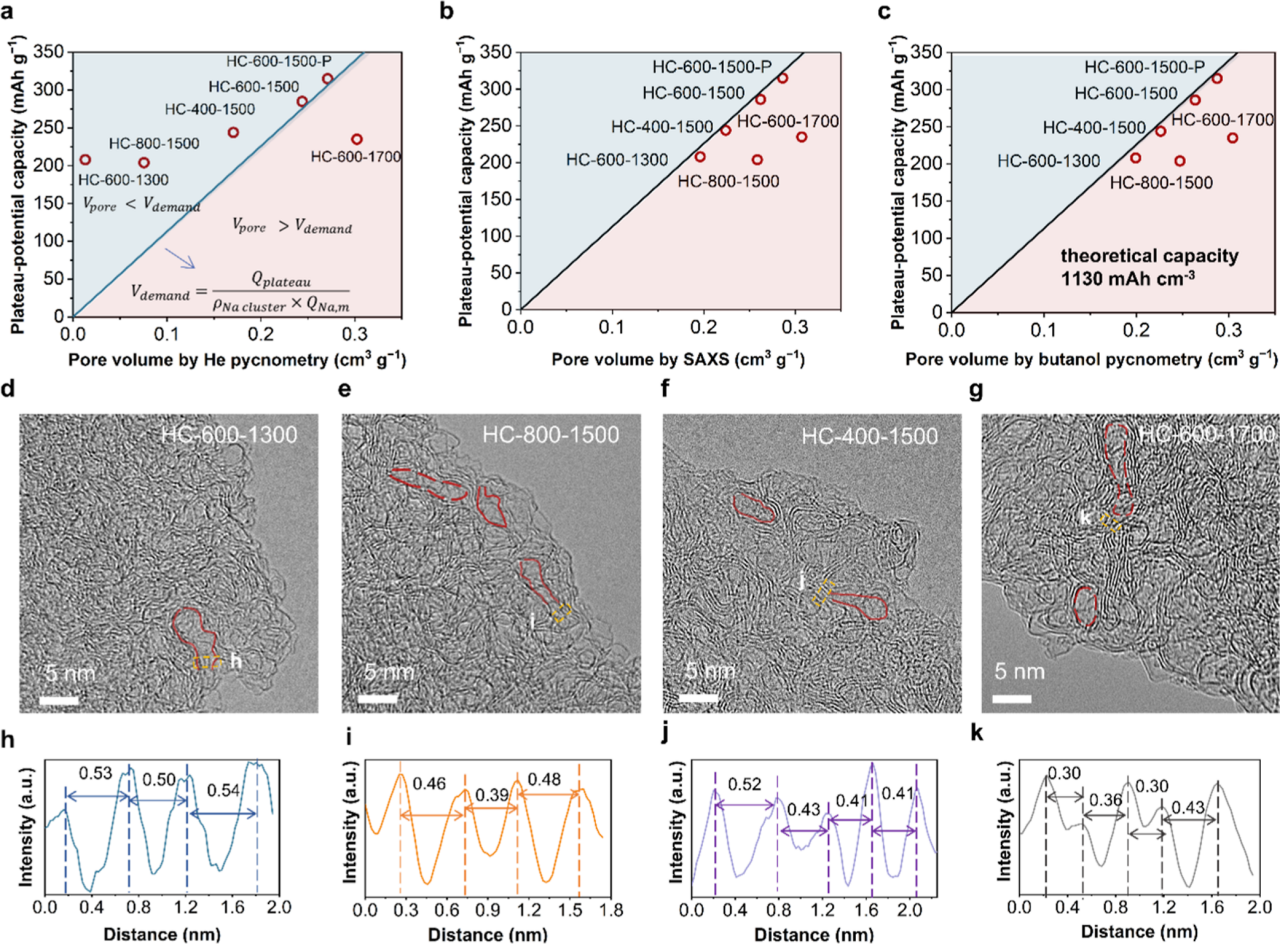
Relationship between PPC and pore volumes determined by (a) helium pycnometry, (b) SAXS, and (c) butanol pycnometry. The diagonal dash line in the panel (a–c) is calculated from $V_{demand}=\frac{Q_{plat}}{\rho_{Na}\times Q_{Na,m}}$ , where *Q*_plat_ is the PPC, ρ_Na_ is the sodium cluster density (0.968 g cm^–3^), and *Q*_Na,m_ is the theoretical capacity of sodium metal (1166 mA h g^–1^). The light blue and light red colors signify that the required pore volume for the formation of sodium clusters is larger and smaller than the pore volume obtained by helium pycnometry, SAXS, and butanol pycnometry, respectively. The deviation of HC-800–1500 and HC-600–1700 from the diagonal line is ascribed to that sodium clusters in large nanopores require large energy barriers and a dense graphene layer that would enable low accessibility of Na^+^ to the nanopores. HR-TEM images of (d) HC-600–1300, (e) HC-800–1500, (f) HC-400–1500, and (g) HC-600–1700. The interlayer spacing in the corresponding region of HR-TEM for (h) HC-600–1300, (i) HC-800–1500, (j) HC-400–1500, and (k) HC-600–1700.

The helium molecule has a size of 0.264 nm, significantly smaller than the solvent molecules of electrolytes such as ethylene carbonate (EC, 0.342 nm) and dimethyl carbonate (DMC, 0.441 nm).^47–50^ The detected localized interlayer spacings of HC-600–1300, HC-800–1500, and HC-400–1500 ranged from 0.4 to 0.54 nm (Figure 5d–f,h-j). The large interlayers between graphenes could be considered as the entrance of the open nanopore.^33^ The EC solvent molecule has a molecular dynamics size of 0.57 nm^27^, therefore, nanopores with aperture diameters between 0.4 and 0.54 nm are accessible to helium but inaccessible to the solvent molecules of the electrolyte. In such open nanopores, Na^+^ ions could acquire electrons to form quasi-metallic sodium clusters.^22^ Therefore, the helium pycnometry underestimates the volume of nanopores in which sodium clusters could form. Notably, both HC-600–1500 and HC-600–1500 P exhibit open nanopores with nanopore aperture diameters ranging from 0.4 to 0.55 nm (Figures S20 and S22), resulting in the underestimation of the closed pore volumes of HC-600–1500 and HC-600–1500 P.

SAXS was utilized to assess the volume of open and closed nanopores. The pore scattering per unit mass was calculated by normalizing the SAXS data for absolute intensity, enabling the calculation of nanopore volumes in HC.^51^ The nanopore volumes for HC-600–1300, HC-800–1500, and HC-400–1500 were 0.196, 0.259, and 0.224 cm^3^ g^–1^, respectively (Table 1 and S15). The pore-filling ratios, derived from these nanopore volumes, were 93.9%, 96.5%, 67.7%, 97.0%, 96.4%, and 69.8% for HC-600–1300, HC-600–1500, HC-600–1700, HC-600–1500 P, HC-400–1500, and HC-800–1500, respectively (Table 1). These results suggest that the nanopore volume of HC was sufficient to accommodate the required pore volume of sodium clusters (Figure 5b). Although SAXS could estimate the nanopore volumes of HC, it could not differentiate the nanopores accessible and inaccessible to the solvent molecules of the electrolyte.

The true density measurements of HC were conducted using butanol pycnometry with a specifically designed pycnometer (methods shown in the experimental section in Supporting Information). The principle of butanol pycnometry aligns with that of helium pycnometry, despite the difference in the filling medium. Butanol possesses a molecular size akin to that of the solvent molecules of electrolyte.^52^ eq 1 shows the method for calculating the nanopore volume by true density. Therefore, the volume of nanopores determined by butanol pycnometry could be approximated as the volume of nanopores that are inaccessible to the solvent molecules of the electrolyte. The pore-filling ratios, obtained from the volume of nanopore inaccessible to the solvent molecules of electrolyte, for HC-600–1300, HC-600–1500, HC-600–1700, HC-600–1500 P, HC-400–1500, and HC-800–1500 were 92.3%, 96.0%, 68.3%, 96.9%, 95.6%, and 76.4%, respectively (Table 1). The nanopore volumes obtained from butanol pycnometry were comparable to those determined by SAXS (Figure 5c). This enables the fast screen of HCs capable of sodium-ion storage and the possibility for prediction of their capacities.

The nanopore volume of HC-800–1500, as determined by butanol pycnometry, was smaller than the nanopore volume calculated by SAXS (Table 1). This discrepancy is attributed to the presence of open pores ranging from 0.9 to 1.3 nm and 1.8 to 3 nm in the HC-800–1500 (Figure S3b). Since these open nanopores are accessible to butanol molecules, butanol pycnometry could not quantify the volume of these nanopores. For HC-800–1500, the volume of nanopore accessible to the solvent molecules of electrolyte was intuitively determined as the difference between the pore volume measured by SAXS and that calculated by butanol pycnometry. This pore volume of HC-800–1500 was approximately 0.022 cm^3^ g^–1^ (Table S17), close to its open micropore volume determined by N_2_ adsorption/desorption (0.029 cm^3^ g^–1^) (Table S2). This nanopore volume accounted for 8.5% of the total nanopore volume of HC-800–1500, which should offer 25 mA h g^–1^ of PPC. The total nanopore volume of HC-800–1500 estimated by SAXS was 0.259 cm^3^ g^–1^ (Table 1), which should offer a specific capacity of 293 mA h g^–1^. However, the actual PPC capacity of HC-800–1500 with a cutoff potential of the sodium deposition was 238 mA h g^–1^ (Figure S14). The difference between the actual PPC and the theoretical PPC was 55 mA h g^–1^, which was attributed to the pores that cannot be filled by sodium clusters. In particular, the PPC offered by the nanopores accessible to the electrolyte of HC-800–1500 accounted for 45.5% of this difference. The other reason for the difference between the actual PPC and the theoretical PPC should be the restricted sodium diffusion in the GNDs. In short, the PPC originates from the filling of quasi-metallic sodium clusters into nanopores that are accessible to sodium ions but inaccessible to the solvent molecules of electrolytes.

The actual pore-filling ratios were determined through ex-situ SAXS. The pore-filling ratios were also calculated by dividing the sodium cluster volume (calculated from the actual PPC) by the nanopore volume. The actual pore-filling ratios and the pore-filling ratios calculated exhibited congruence (Table 1 and S10). This is a quantitative relationship to suggest that the PPC of HC is attributed to the filling of quasi-metallic sodium clusters within the nanopore. A correlation was observed between the nanopore volume and the actual PPC (Figure 5c). The theoretical volumetric specific capacity of sodium was 1130 mA h cm^–3^. Therefore, the relationship between the theoretical PPC and pore volume of HC detected should be given by eq 4

4 $$C_{T,plat}=V_{pore}\times 1130$$

where *C*_T,plat_ (mA h g^–1^) is the theoretical PPC and *V*_pore_ is the volume of open and closed pores that could store sodium clusters ideally. The deviation of actual PPC from the above theoretical relationship should be ascribed to the nonideal pore architecture that could not be fully filled with sodium clusters.

HC-800–1500 and HC-600–1700 had localized interlayer spacing ranging from 0.30 to 0.36 nm observed by HRTEM images (Figures 5g,k, and S24d–f). The intercalation of Na^+^ in graphene layers with smaller than 0.349 nm of interlayer spacing is energetically unfavorable.^53^ Although the diffusion of Na^+^ GNDs with interlayer spacings from 0.349 to 0.36 nm is permissible, the diffusion process of Na^+^ is sluggish.^54^ The narrow interlayer spacing hinder the diffusion of Na^+^ ions toward the nanopores, preventing the storage of sodium clusters within pores. The lower pore-filling ratios in HC-800–1500 and HC-600–1700 compared to other HCs are a consequence of the inaccessibility of some nanopores due to the dense graphene layer structure and the partial filling of large nanopores. As a result, the PPC and pore volume values tested by SAXS and butanol pycnometry for the HC-800–1500 and HC-600–1700 deviate from the theoretical linear relationship (Figure 5b,c).

Scheme 3 shows the influence of the carbonization regime on the pore architecture and the influence of pore architecture on the storage of sodium ions. The HC-600–1300 possessed GNDs with short *L*_a_ and *L*_c_ and a large *d*_002_. This particular GND structure resulted in the nanopore in HC-600–1300 being almost accessible to helium but inaccessible to the solvent molecules of the electrolyte (Scheme 3). HC-400–1500 and HC-600–1500 possessed partially open nanopores accessible to helium but inaccessible to the solvent molecules of the electrolyte. The HC-800–1500 exhibited wide-aperture pores accessible to the solvent molecules of electrolyte, and most of these pores were accessible to helium. These wide-aperture pores prevent the storage of sodium clusters. Both HC-800–1500 and HC-600–1700 displayed denser graphene layers with large GNDs and large nanopores, which could impede the Na^+^ diffusion through the graphene layer and restrict the growth of sodium clusters, consequently lowering the pore-filling ratios.

**Scheme 3:**
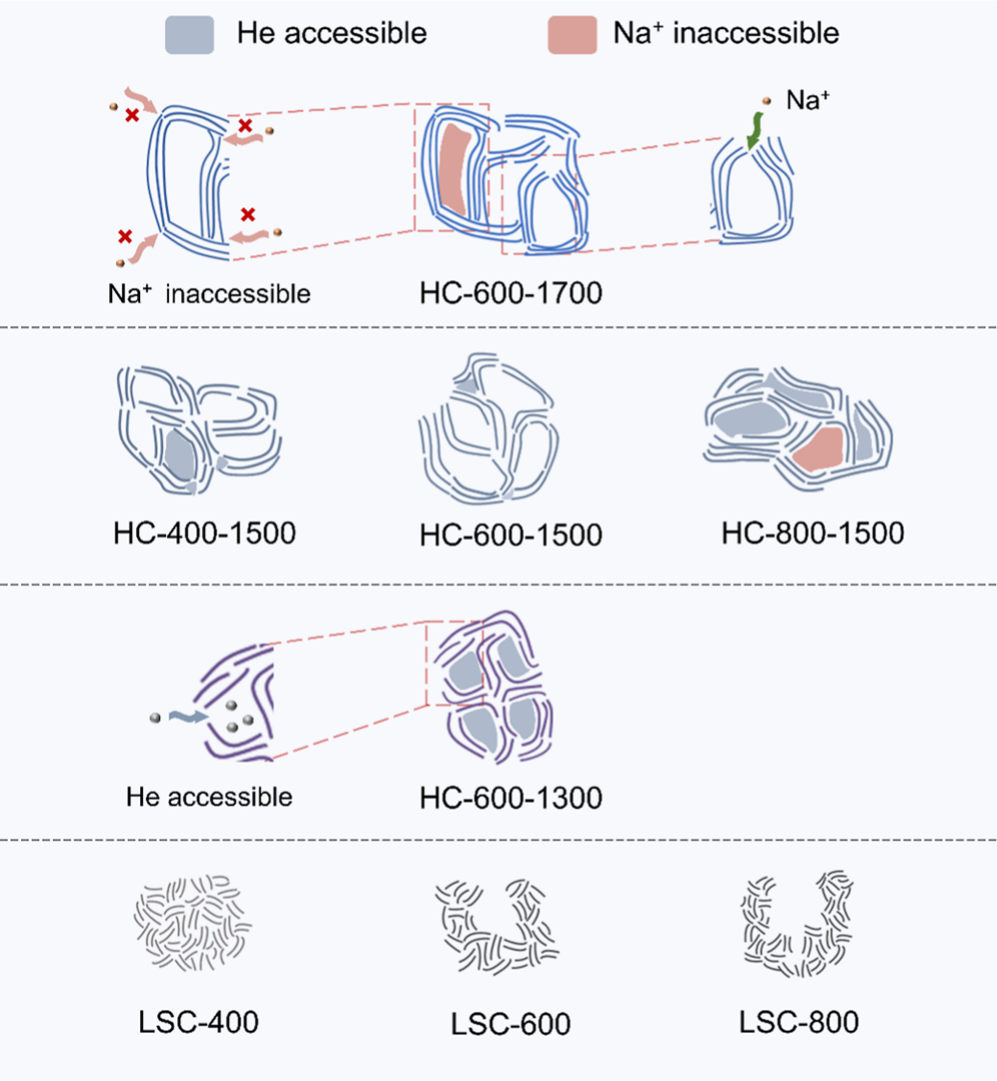
Schematic Illustration of the Pore Architecture Properties of HC. Due to the Smaller Size of the Sodium Ion (0.204 nm) than the Helium Molecule (0.264 nm), the Pores Accessible to Helium Should Also be Accessible to Sodium Ions

Additionally, we estimated the pore volume of HCs derived from the well-known precursors through butanol pycnometry, aiming to confirm its applicability in estimating the volume of nanopores inaccessible to the solvent molecules of electrolyte. The volume of HC, which includes the volume of GNDs and nanopores inaccessible to the solvent molecules of electrolyte, was calculated by dividing the mass of the expelled butanol in the pycnometer by the density of butanol (the detailed experimental process is shown in Supporting Information). The density of HC (ρ_HC_) could be determined by eq 5

5 $$\rho_{HC}=\frac{M_{HC}\times \rho_{butnol}}{M_{HC}+M_{2}-M_{3}}$$

where *M*_HC_ represents the mass of HC, ρ_butanol_ is the density of butanol, *M*_2_ is the mass of the pycnometer filled with butanol, and *M*_3_ is the mass of the pycnometer filled with butanol and HC (Figure 6a). Various HC samples denoted as EHL-HC, G-HC, HG-HC, B-HC, C-HC, and AL-HC were synthesized by the carbonization of enzymatically hydrolyzing lignin (EHL), glucose, hydrothermal glucose, bamboo, cellulose, and alkali lignin (AL) at temperatures of 1300 or 1500 °C. The heat treatment temperatures of 1300 and 1500 °C were chosen because the d_002_ of the HCs prepared at these temperatures was above 0.37 nm and the *L*_a_ values were between 3 and 6 nm (Table S18). These microcrystalline structures enable sodium ions to enter most of the nanopores, rendering the nanopores highly accessible to sodium ions.

**Figure 6:**
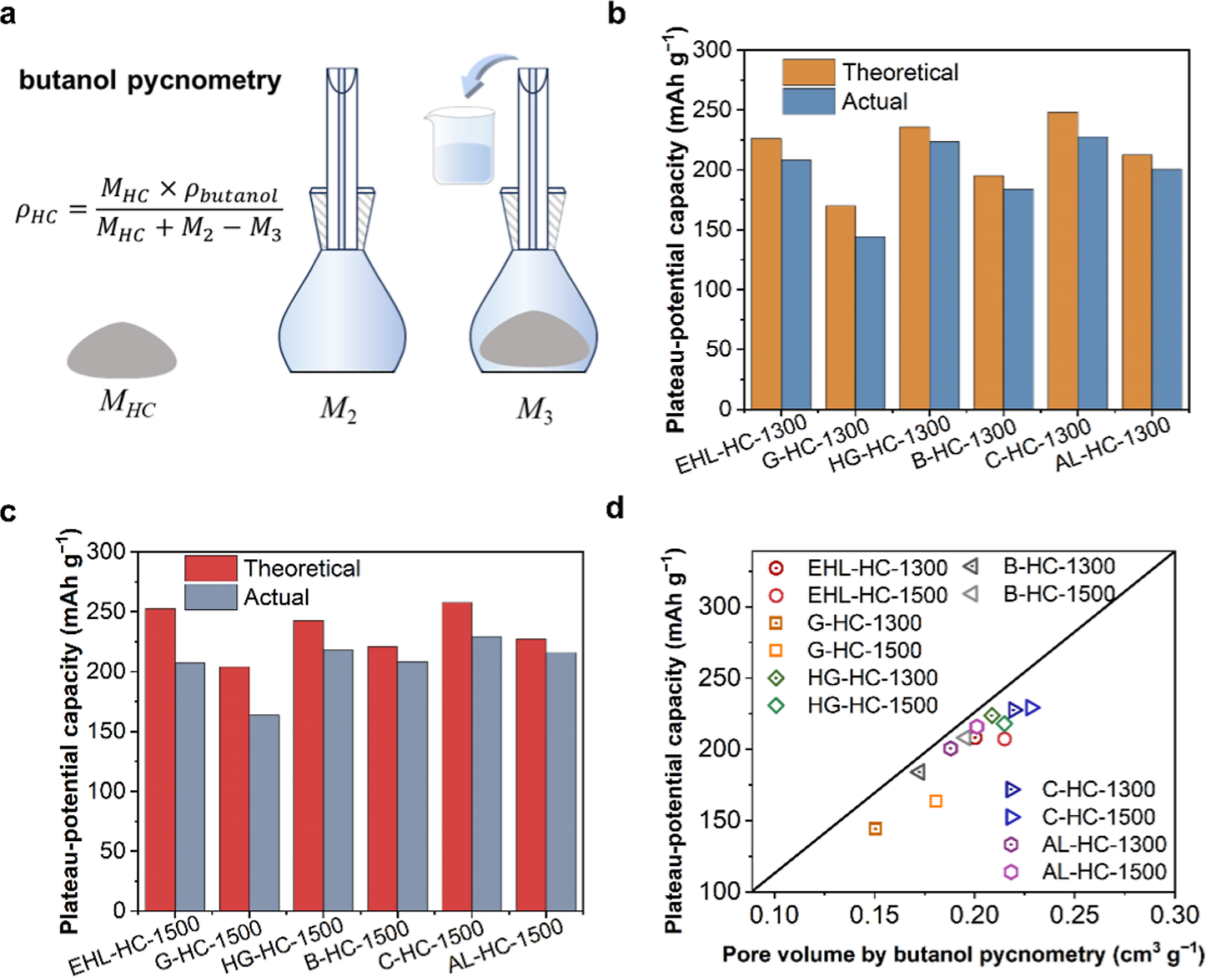
(a) Schematic illustrating the true density measurement by butanol pycnometry. Theoretical and actual PPCs of (b) B-HC-1300, B-HC-1500, C–HC–1300, C–HC–1500, AL–HC–1300, AL–HC–1500, (c) EHL–HC–1300, EHL–HC–1500, G–HC–1300, G–HC–1500, HG–HC–1300, and HG–HC–1500 at a current density of 20 mA g^–1^. The theoretical PPCs of HCs were determined by multiplying the pore volume obtained through the butanol pycnometry by the theoretical volumetric specific capacity of sodium (1130 mA h cm^–3^). (d) Relationship between PPC and pore volume measured by butanol pycnometry.

The theoretical PPCs of HCs were determined by multiplying the pore volume obtained through the butanol pycnometry by the theoretical volumetric specific capacity of sodium (1130 mA h cm^–3^). The experimentally measured PPCs of HC were consistently lower than the theoretical PPC (Figure 6b,c). The pore volumes of EHL-HC, G-HC, HG-HC, B-HC, C-HC, and AL-HC could all fulfill the pore volume requirements for the formation of quasi-metallic sodium clusters (Figure 6d). Moreover, we probe the mechanism of sodium-ion storage behavior in YP-50F porous carbon with butanol pycnometry. The true density of YP-50F porous carbon was tested as 2.199 g cm^–3^ (Table S16). The PPC of YP-50F porous carbon was 0 mA h g^–1^ (Figure S26). The nanopores of YP-50F porous carbon were almost open pores accessible to the solvent molecules of electrolytes, thus incapable of storing sodium clusters (Figure S27). In conclusion, the butanol pycnometry could fast screen HC anodes and predict their PPCs for the storage of sodium ions in the open and closed pores inaccessible to the solvent molecules of electrolytes.

## 3 Conclusions

In conclusion, we successfully manipulated the pore architecture of HC by employing a self-templating method. The potential-dependent sodium-ion storage mechanism in HC was elucidated as a sequence of “adsorption, adsorption/intercalation, and pore-filling”. The PPC arises from the insertion of quasi-metallic sodium clusters into nanopores that are accessible to Na^+^ ions but impermeable to electrolyte solvent molecules.

Helium pycnometry, SAXS, and butanol pycnometry method were employed to measure the nanopore volume filled with sodium clusters. However, helium pycnometry tends to underestimate the nanopore volume available for sodium cluster formation due to the small size of helium molecules. The volume of nanopores where sodium clusters form can be more accurately estimated using SAXS and butanol pycnometry.

A direct correlation was established between the nanopore volume detected by butanol pycnometry and the actual PPC. The actual PPC deviation from the direct correlation stems from two main factors. First, sodium clusters are unable to fill nanopores larger than 4 nm and only partially fill those larger than 2 nm, resulting in a reduced apparent pore-filling ratio. Second, GNDs with small interlayer spacing and extended crystalline planes can hinder sodium ion diffusion, limiting access to closed nanopores. Notably, HCs prepared at high temperatures (exceeding 1500 °C) exhibit decreased specific capacities due to the enlargement of closed pores and the extensive growth of GNDs. Our proposed butanol pycnometry method not only predicts the theoretical PPC of HC but also serves as a rapid screening tool for selecting HC precursors and forecasting the PPCs of the resulting HCs.

## 4 Methods

### 4.1 Materials

Sodium lignosulfonate (LS) was purchased from Chempack Co., Ltd. in Russia. The LS contains 2.67 mmol g^–1^. The sulfonate group content of LS was measured by the burning method. A certain mass of LS was burned in a muffle furnace at 600 °C for 2 h. Alkali lignin (AL) and enzymatic hydrolysis lignin (EHL) were purchased from Longlive Biotechnology (Shandong province, China). Butanol, bamboo powders, cellulose, and glucose were purchased from Aladdin Chem. Polytetrafluoroethylene (PTFE), polyvinylidene difluoride (PVDF) binder, *N*-methylpyrrolidone (NMP), carbon black, and commercial YP-50F activated carbon from Kuraray Chemical Industry, Japan, were purchased from Guangdong Canrd New Energy Technology Co., Ltd. All other chemical reagents were used without further purification.

### 4.2 Preparation of HCs

LS was calcined at 400, 600, and 800 °C in a tubular furnace with a constant N_2_ flow (60 sccm) under a heat ramping rate of 5 °C min^–1^ for 1 h. The sintered products were soaked in 6 wt % HCl solution to remove impurities. The obtained porous carbons were washed with deionized water to a pH value close to 7 and dried at 90 °C for 12 h. The as-prepared carbons were denoted as LSC-400, LSC-600, and LSC-800 (400, 600, and 800 were carbonization temperatures). LSC-400, LSC-600, and LSC-800 were heat-treated at high temperatures (1300, 1500, and 1700 °C) in a tubular furnace with a constant N_2_ flow (60 sccm) under a ramp rate of 5 °C min^–1^ for 2 h to prepare HC samples. The as-prepared carbons were denoted as HC-400–1500, HC-600–1500, HC-800–1500, HC-600–1300, and HC-600–1700 (HC-X-Y: X represents the preparation temperature of LSC and *Y* represents the heat treatment temperature of HC). For the preparation of HC-600–1500 P sample, 0.05 g of pitch was dissolved in 5 mL of tetrahydrofuran and 1 g of LSC-600 was added to the solution forming a suspension. Air bubbles were removed from the suspension by sonication and centrifugation. The suspension was then placed at room temperature to allow the tetrahydrofuran to evaporate completely. The obtained solid was placed in a tube furnace for heat treatment at 1500 °C for 2 h under N_2_ protection. The resulting HC was denoted as HC-600–1500 P.

Five g of glucose was dissolved in 50 mL to obtain the precursor solution. Subsequently, the solution was hydrothermally treated at 190 °C for 8 h to prepare the precursor of HC. The precursor of HC was denoted HG. The AL, EHL, bamboo, cellulose, glucose, and HG were carbonized at 1300 and 1500 °C in a tubular furnace with a constant N_2_ flow (60 sccm) under a ramp rate of 5 °C min^–1^ for 2 h. The HCs derived from AL, EHL, bamboo, cellulose, glucose, and HG were denoted as AL–HC–1300, AL–HC–1500, EHL–HC–1300, EHL–HC–1500, B-HC-1300, B-HC-1500, C–HC–1300, C–HC–1500, G–HC–1300, G–HC–1500, HG–HC–1300, and HG–HC–1500 (B represents bamboo, C represents cellulose, G represents glucose, HG represents the hydrothermally treated glucose, and 1300 and 1500 are the heat treatment temperatures).

### 4.3 Electrode Preparation

The HC electrodes were prepared by mixing the HC samples with carbon black and PVDF at a weight ratio of 8:1:1 in NMP solvents (mechanically mixed for at least 10 h) and casting on Cu foil with the blade coating technique. After drying at 80 °C under vacuum for 12 h, the electrodes were cut into discs with a diameter of 12 mm. The mass loadings of electrodes were around 1.5 mg cm^–2^. Half cells (CR2032) were assembled with sodium metal foil (99.8%, Aladdin, China) as the counter/reference electrode, a glass-fiber separator (Whatman), and 1 M sodium hexafluorophosphate (NaPF_6_) dissolved in ethylene carbonate and diethyl carbonate (EC/DEC, equal volume) as an electrolyte. All the assembling operations were conducted in the argon-filled glovebox.

### 4.4 Material Characterization

#### 4.4.1 Structural Analysis

The defect degrees of HCs were characterized by the LabRAM HR Evolution microconfocal Raman spectrometer (Raman, HORIBA Jobin Yvon, France). X-ray diffraction patterns were collected on a D8 Advance X-ray diffractometer (XRD, Bruker, Germany) with Cu Kα radiation (λ = 1.5406 Å). The chemical compositions of the HCs samples were analyzed by Escalab 250Xi X-ray photoelectron spectroscopy (XPS, Thermo Fisher Scientific, USA). The elemental content of the HC samples was analyzed by the EA-3000 elemental analyzer (EA, Eurovector, Italy). The signal of sodium clusters in HCs was performed by AVANCE III HD 600 MHz magic angle spinning (MAS) solid-state nuclear magnetic resonance (NMR) experiments (MAS ssNMR, Bruker, Germany). The microstructures of the HCs samples were investigated by a Talos F200S transmission electron microscopy (TEM, Thermo Fisher Scientific, USA). The pore structures of HC were analyzed by Xeuss 3.0 small-angle X-ray scattering (SAXS, Xenocs, France) with a monochromatized and parallel Cu Kα X-ray of 30 W. The ex-situ SAXS and wide-angle X-ray scattering (WAXS) were collected on the BL19U2 of the Shanghai Synchrotron Radiation Facility. The specific surface areas and pore diameters were measured at 77 K using ASAP 2460 N_2_ adsorption/desorption (Micromeritics, USA) and 273 K using ASAP 2020 CO_2_ adsorption/desorption (Micromeritics, USA). True densities of HCs were measured via helium gas pycnometry with the AccuPyc-1345 density analyzer (Micromeritics, USA) and *n*-butanol pycnometry with a pycnometer (Guangdong Hongtuo Instrument Technology Co., China). The SAXS data were analyzed using the SasView 4.2.2 ^55^ and Igor Pro 9.02 software.^56^ The semiempirical Teubner–Strey model with SAXS was used to analyze the pore structure of HC. The neutron total scattering data for HC were collected in a multi physics instrument (MPI) at the China Spallation Neutron Source (CSNS).^57,58^ The 800 ∼1000 mg of HC powder samples were placed in a 9 mm TiZr cell and measured at room temperature. HC-600–1300, HC-600–1500, HC-600–1500 P, and HC-600–1700 were measured for 4 h. HC-400–1500 and HC-800–1500 were measured for 3 h. The neutron total scattering data of HC were analyzed by the Mantid software and Fourier transformation to obtain the pair distribution functions (PDF) with *Q*_max_ = 50 Å^–1^.

#### 4.4.2 In situ and ex-situ experiments

The electrodes for in situ Raman, ex-situ SAXS, and ex-situ XRD measurements were prepared by mixing HC sample and PVDF binder in NMP solvents with a weight ratio of 9:1 to remove the effect of carbon black. The mass loadings of electrodes were around 2 mg cm^–2^. The electrodes for ex-situ ^23^Na MAS ssNMR were prepared by mixing HC sample, carbon black, and PVDF at a weight ratio of 8:1:1 in ethanol solvents. The mass loadings of electrodes were around 3 mg cm^–2^.

The electrodes for the ex-situ SAXS, ex-situ ^23^Na MAS ssNMR, and ex-situ XRD were collected under constant current at a current density of 20 mA g^–1^ and constant voltage at the designated potential for 3 h. The electrodes of the disassembly were cleaned several times using the DEC solvent before the measurement. The electrodes were then dried in an argon-filled glovebox to remove the surface solvent. The electrodes for in situ Raman were tested under constant current at a current density of 20 mA g^–1^.

In ex-situ ^23^Na MAS ssNMR analysis, the NMR samples were sealed in a 4 mm Φ sample rotor. Ex situ ssNMR experiments were performed at a MAS frequency of 12 kHz, and the signals were collected by accumulating 3200 scans. The ^23^Na chemical shifts were referenced to NaCl (1 M a.q.) as 0 ppm.

ASSOCIATEDCONTENT.

## Supplementary Material



## Notes

Discussions of supporting experimental section, detailed calculation of A_D_/A_G_ values from Raman spectra, detailed data analysis of XRD, detailed data analysis process of SAXS, pore parameters, elemental composition, crystalline structural parameters, true density, TEM, and electrochemical behaviors (PDF)
