# Supplementary material for: Elucidation of the Sodium-Ion Storage Behaviors in Hard Carbon Anodes through Pore Architecture Engineering
Source: ACS Nano. 2025 Jun 9;19(24):22201–16. doi: 10.1021/acsnano.5c03700 (PMC12203633; doi:10.1021/acsnano.5c03700)
Supplement: Supplementary file 1 [file nn5c03700_si_001.pdf]

Supporting Information for

Elucidation of the sodium-ion storage behaviors in hard carbon anodes through pore architecture engineering

Wenbin Jian, Xueqing Qiu, Huaican Chen, Jian Yin, Wen Yin, Husam N. Alshareef* and Wenli Zhang**

[1]Guangdong Provincial Key Laboratory of Plant Resources Biorefinery, School of Chemical Engineering and Light Industry, Guangdong University of Technology (GDUT), 100 Waihuan Xi Road, Panyu District, Guangzhou 510006, China.

[2]Guangdong Provincial Laboratory of Chemistry and Fine Chemical Engineering Jieyang Center, Jieyang, 515200, China.

[3]Center for Renewable Energy and Storage Technologies (CREST), King Abdullah University of Science and Technology (KAUST), Thuwal 23955-6900, Saudi Arabia.

[4]Laboratory of Environmental Sciences and Technology, Xinjiang Technical Institute of Physics & Chemistry, Chinese Academy of Sciences, Urumqi 830011, China.

[5]Institute of High Energy Physics, Chinese Academy of Sciences, Beijing 100000, China.

[6]Spallation Neutron Source Science Center, Dongguan 523000, China.

1. Supporting Experimental Section

1.1 Density measurement by n-butanol pycnometry

A sample of HC ranging from 1 g to 1.5 g was prepared. The mass of HC was weighed accurately using a balance with an accuracy of 0.01 mg and recorded as $\boldsymbol{M}_{\mathbf{HC}}$. The butanol was poured into a pycnometer of approximately 50 mL. The pycnometer is made of borosilicate glass and has a tolerance of 0.008 mL. The mass of the butanol-filled pycnometer was weighed as $\boldsymbol{M}_{\mathbf{2}}$. The butanol inside the pycnometer was poured out 2/3, and all the weighed HC was poured into the pycnometer. The pycnometer containing HC and butanol was ultrasonicated until there were no air bubbles in the butanol. The pycnometer was placed at room temperature until the HC was completely deposited at the bottom of the pycnometer. Then, butanol was filled to the inside of the pycnometer, and the mass of the pycnometer at this point was weighed and recorded as $\boldsymbol{M}_{\mathbf{3}}$. The true density of HCs at this point could be calculated by the following equation:

$$\rho_{\mathrm{HC}}=\frac{M_{\mathrm{HC}}\times\rho_{\mathrm{butanol}}}{M_{\mathrm{HC}}+M_2\text{-}M_3} \qquad \text{(S1)}$$

where ρ_{butanol} is the density of butanol. The pore volumes of HC were calculated from the equation:

$$V_{\mathrm{pore}}=\frac{1}{\rho_{\mathrm{HC}}}-\frac{1}{\rho_{\mathrm{struc}}} \qquad \text{(S2)}$$

where V_{pore} is the pore volumes of HC, ρ_{HC} is the density of HC measured by butanol pycnometry, and ρ_{struc} is the structural density of HC.

The ρ_{struc} is obtained by the equation：

$$\rho_{\mathrm{struc}}=\rho_{\mathrm{graphite}}\frac{d_{002}^{\mathrm{graphite}}}{d_{002}}\left(\frac{d_{100}^{\mathrm{graphite}}}{d_{100}}\right)^2 \qquad \text{(S3)}$$

ρ_{graphite} is the density of graphite. d_{002} and d_{100} are the (002) interlayer spacing and the (100) interlayer spacing for HC, respectively. $d_{002}^{\mathrm{graphite}}$ and $d_{100}^{\mathrm{graphite}}$ are the d_{002} and d_{100} of graphite.

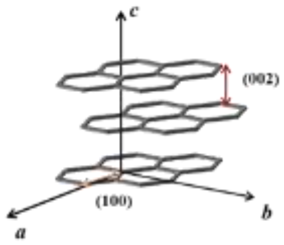

The (002) crystalline plane of HC corresponds to the *c*-axis direction of the graphite lattice. The (100) crystalline plane corresponds to the *a*-axis direction of the graphite lattice. The increase or decrease of the d_{002} changes the thickness of the graphite lattice. The increase or decrease of d_{002} changes the volume of the lattice in the one-dimensional direction. While the increase or decrease of d_{100} changes curved surface of the *a*-axis direction of the graphite lattice. The increase or decrease of d_{100} changes the lattice volume in the two-dimensional direction. Therefore, the ρ_{struc} is inversely proportional to d_{002} and inversely proportional to the square of d_{100}.

1.1 Electrochemical tests

The galvanostatic charge/discharge (GCD) tests were conducted on a BTS4008-5V-20mA Neware battery test system (Neware, Shenzhen, China) at room temperature. The cyclic voltammetry (CV) and electrochemical impedance spectroscopy (EIS) measurements were conducted on a VMP3e and DH7000 electrochemical workstation (Bio-Logic, France, and DongHua Test, China).

There is a power-law relationship between the CV scan rate (v) and the peak current (I):

$$I = av^{b} \qquad \text{(S4)}$$

The *b*-value is calculated by converting the equation (S5) to

$$logI = blogv + \log a \qquad \text{(S5)}$$

where i is the peak current, mA; v is the scan rate, mV s^{-1}; a and b are adjustable values.

Note S1. Detailed calculation of A_D/A_G values from Raman

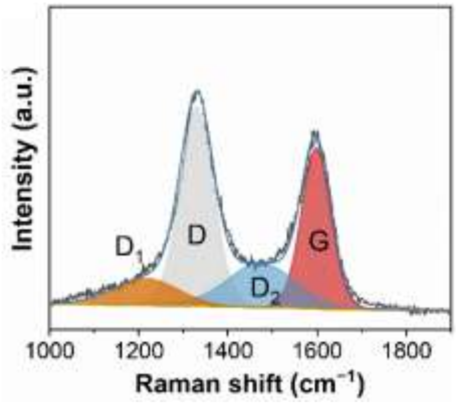

Figure S1.1 Raman spectra for HC.

The Raman spectra of HCs can be divided into four sub-peaks: (1) the D band at ~1330 cm^{-1} corresponding to the A_{1g} symmetry vibrations of disordered carbon atoms in the defective graphitic structure; (2) the D_1 band at ~1210 cm^{-1} attributed to the heterostructure at the termination edge of sp^2-sp^3 graphene or to the A_{1g} vibration of C-C/C=C; (3) the D_2 band at ~1475 cm^{-1} peak ascribed to the vibration of the short-range sp^3 amorphous carbon; (4) the G band at ~1600 cm^{-1} corresponding to the E_{2g} symmetry vibration of sp^2 carbon atoms[1, 2].

The A_{Dx}/A_G value is calculated by dividing the D_x band peak area by the G band peak area.

Note S2. Detailed data analysis of XRD

The interlayer spacing of (002) and (100) crystalline planes is calculated by the Bragg equation:

$$2dsin\theta = n\lambda \qquad \text{(S6)}$$

where d is interlayer spacing, θ is half of peak center 2θ, and λ is the wavelength of the X-ray (0.154 nm).

The crystalline length (L_a) and thickness (L_c) of HC could be calculated by the Scherrer equations:

$$L(\text{nm}) = \frac{K\lambda}{\beta\cos\theta} \qquad \text{(S7)}$$

where K values of 1.84 and 0.90 are for the (100) and (002) peaks of the carbon materials, respectively, and β is the full-width half maximum (FWHM) of the XRD peak. The FWHM of L_c and L_a is calculated using the FWHM of the diffraction peak of the (002) crystalline plane and the (100) crystalline plane, respectively.

The number of graphene layer stacks (N) is calculated by the following equation:

$$N = \frac{L_c}{d_{002}} + 1 \qquad \text{(S8)}$$

Note S3. Absolute intensity calibration of SAXS data[3]

HCs are composed of randomly arranged stacked graphene nanodomains and abundant nanopores. HCs exhibit pore-scattering shoulders in the range of scattering vectors Q from 0.08 to 0.8 $Å^{-1}$. The pore size information of HC could be obtained by fitting the SAXS curve of HC. If it is necessary to calculate the pore volume fraction of HC by SAXS, the absolute intensity of the SAXS signal must be calibrated. We used glassy carbon as a standard SAXS sample (NIST SRM 3600, SN: D28) to calibrate the scattering intensity of HC.

The HC sample is a powder sample and the glassy carbon is a bulk sample. There is a gap between the powder samples. And the thickness between HC and glassy carbon is not consistent. Therefore, transmission and thickness calibration of HC samples are performed.

$$I_{d,T}(Q)=\frac{I_{HC}(Q)\times T_{HC}}{d_{HC}} \qquad \text{(S9)}$$

$$SF=\frac{I_{abs,GC}(Q)}{I_{GC}(Q)} \qquad \text{(S10)}$$

$$I_{abs,HC}(Q)=\frac{I_{d,T}(Q)\times SF\times d_{GC}}{T_{GC}} \qquad \text{(S11)}$$

Where $I_{d,T}(Q)$ is the normalized intensity for the thickness (d_{HC}) and the transmission coefficient (T_{HC}) of the HC sample. SF is the calibration factor. $I_{abs,GC}(Q)$ and $I_{GC}(Q)$ are the absolute and experimental intensities, respectively. $I_{abs,HC}$ is the absolute intensity of the HC. d_{GC} is the thickness of the glassy carbon, and T_{GC} is the transmission coefficient of the glassy carbon.

$I_{abs,HC}(Q)$ is the volumetric scattering cross-section of the sample with units of cm^{-1}. The specific mass scattering cross-section $I^{cm^2\ g^{-1}}$ can be obtained by normalizing the volumetric scattering cross-section $I^{cm^{-1}}$ by effective bulk density ρ_{eff}. The ρ_{eff} of a powder sample at the illuminated spot is determined based on the transmission of the sample, which takes into account the specific attenuation factor for carbon μ/ρ[4]:

$$I^{\mathrm{cm2\ g\text{-}1}}(\mathrm{Q})=\frac{I^{\mathrm{cm\text{-}1(Q)}}}{\rho_{\mathrm{eff}}} \qquad \text{(S12)}$$

$$\rho_{\mathrm{eff}}=\frac{\mathrm{In}T_{HC}}{\mu/\rho\cdot d_{HC}} \qquad \text{(S13)}$$

Here, μ/ρ is the X-ray mass attenuation coefficient of the sample. The μ/ρ = 4.51 and 1.37 $cm^2\ g^{-1}$ have been determined by interpolation of the NIST data base at the photon energy corresponding to Cu K-α (8.04 keV) and synchrotron radiation source (12.00 keV), respectively.

Note S4. Detailed data analysis process of SAXS

The SAXS curves at scattering vectors (Q) lower than 1 $Å^{-1}$ can be divided into three parts: (1) a slope in Q^{-n} at Q lower than 0.08 $Å^{-1}$, corresponding to Porod's law for scattering from macroscopic sharp surfaces of powder grains (I_{Porod}); (2) the scattering from micropores in the 0.08 to 1 $Å^{-1}$ Q range (I_{mp}); (3) the background scattering signal ($I_{backgrounds}$):

$$I^{SAXS}(Q)=I_{Porod}+I_{mp}+I_{backgrounds} \qquad (S14)$$

The pore structure of HC is analyzed by the semi-empirical Teubner-Strey model[4]:

$$I_{mp}= I_0\frac{1}{1+C_1Q^2+C_2Q^4} \qquad (S15)$$

Here I_0, C_1 and C_2 are obtained by adjusting the following equations:

$$I_0=\frac{8\pi}{\rho_{struc}}\phi(\Delta SLD)^2\frac{\xi^3}{\left(1+\left(\frac{2\pi\xi}{d}\right)^2\right)^2} \qquad (S16)$$

$$d=2\pi\left[\frac{1}{2}C_2^{-\frac{1}{2}}-\frac{C_1}{4C_2}\right]^{-\frac{1}{2}} \qquad (S17)$$

$$\xi=2\pi\left[\frac{1}{2}C_2^{-\frac{1}{2}}+\frac{C_1}{4C_2}\right]^{-\frac{1}{2}} \qquad (S18)$$

Where ρ_{struc} is the structural density, *SLD* is the contrast of scattering length density. The *SLD* of the carbon material is calculated by the software SasView 4.2.2. The *SLD* of the pores is 0. Δ*SLD* is equal to the *SLD* of the carbon material minus the *SLD* of the pore. *d* is the pore-pore distance, and ξ is the correlation length that limits the extension of the order. The *d* and ξ are finally obtained by fitting SasView 4.2.2.

The average pore size D could be calculated by the following equation:

$$D=2\sqrt{5C_1} \qquad (S19)$$

A parameter f_a, called "amphiphilic factor" for microemulsions[3], could be considered as the connectivity level of HC pore:

$$f_a=\frac{C_1}{2\sqrt{C_2}} \qquad (S20)$$

A larger value of f_a indicates that the pores are more disordered and connected[4].

To determine the nanopore size distribution of HC, the SAXS data were analyzed using the Modeling tool in the Irena package for Igor Pro 9.02 software (by WaveMetrics)[5]. The pore size distribution of HC is modeled by using a basic small angle scattering (SAS) formula:

$$I_{mp}(Q)=|\Delta\rho|^2 \int_0^{\infty}|F(Q, R)|^2 V^2(R)\, N\, P(R)\, dR \qquad \text{(S21)}$$

where $\Delta\rho$ is contrast, $\Delta\rho$=scale($\Delta SLD_{HC} - \Delta SLD_{pore})^2$, $F(Q, R)$ is the scattering form factor, $V(R)$ is the particle volume, N is the total number of scattering particles, $P(R)$ is the probability of occurrence of the pore at the size of R. We assumed that the pores are ellipsoidal and spherical, and the scattering between the pores doesn't interfere with each other. Hence, the form factor is spheroid and the structure factor is a dilute system[6].

For sphere form factors with aspect ratios between 0.99 and 1.01, the scattering form factors are as follows:

$$F(Q, R)=\frac{3(\sin(QR)-(QR)\cos(QR))}{(QR)^3} \qquad \text{(S22)}$$

The particle volume equation is as follows:

$$V(R)=\frac{4\pi R^3}{3} \qquad \text{(S23)}$$

For aspect ratios less than 0.99 and larger than 1.01, the standard form factor for spheroid is used:

$$F(Q, R)=\int_0^{\infty}\frac{3(\sin(QR)-(QR)\cos(QR))}{(QR)^3}d(QR) \qquad \text{(S24)}$$

where $QR=QR*\sqrt{1+(AR^2-1)*\mathrm{CosTh}^2}$, over of CosTh = 0 to 1.

P(R) is the Schulz-Zimm size distribution given by

$$P(R)=\left(\frac{z+1}{R_{avg}}\right)^{z+1}\frac{R^z}{\Gamma(z+1)}e^{(-(z+1)\frac{R}{R_{avg}})} \qquad \text{(S25)}$$

where R_{avg} is the average pore radius, $\Gamma(x)$ is the gamma function, and z is an indicator of the degree of polydispersity associated with the root-mean-square deviation (RMSD), σ, of the pore size distribution. The σ is determined by the following function:

$$\sigma = R_{avg}\sqrt{\frac{1}{z+1}} \quad \text{(S26)}$$

Note S5. Detailed data analysis process of *ex-situ* SAXS

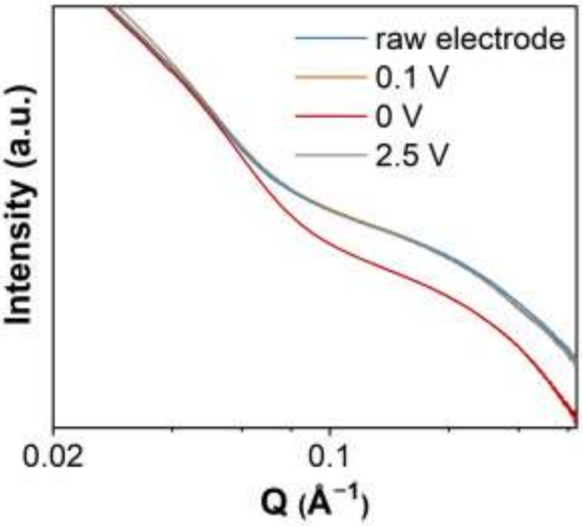

Figure S1.2 *ex-situ* SAXS patterns for HC-600-1300.

The SAXS intensity of the HC-600-1300 electrode at 0.1 V was almost the same as that of the pristine electrode (Figure S1.2), indicating that the density and pore structure of the HC hardly changed. During the pore-filling process of sodium clusters, the decrease in the intensity of I_{mp} arises from the increase of pore density[6-8]. Therefore, the shape parameters of HC are constant, i.e., d, ξ, φ, ρ_{struc}, and SLD_{HC}, and only the density of the pores changes during the filling of sodium clusters. SLD_{pore} and ΔSLD ($\Delta SLD = SLD_{HC} - SLD_{pore}$) are variable. According to Eqs. (S15) and (S16), the pore scattering intensity is proportional to the square of ΔSLD, i.e., $I_{mp} \propto (\Delta SLD)^2$. $\Delta SLD_{HC,\ 0V}$ could be obtained by fitting the SAXS curves of the fully sodiated HC and then calculating the $SLD_{pore,\ 0V}$. The scattering length of Na atoms (SL_{Na}) is 8.22×10^{10} cm g^{-1}. The density of the pore after filling ($\rho_{filled\ pore}$) could be calculated by the equation $\rho_{filled\ pore} = SLD_{pore,\ 0V}/SL_{Na}$. When the pore is completely filled by sodium clusters, $\rho_{filled\ pore}$ is equal to the density of sodium clusters (ρ_{Na} = 0.968 g cm^{-3}), and $SLD_{pore,\ 0V}$ is equal to SLD_{Na} (7.96×10^{-6} Å^{-2}). Therefore, the pore-filling ratio of the nanopore is $\rho_{filled\ pore}/\rho_{Na}$ or $SLD_{pore,\ 0V}/SLD_{Na}$.

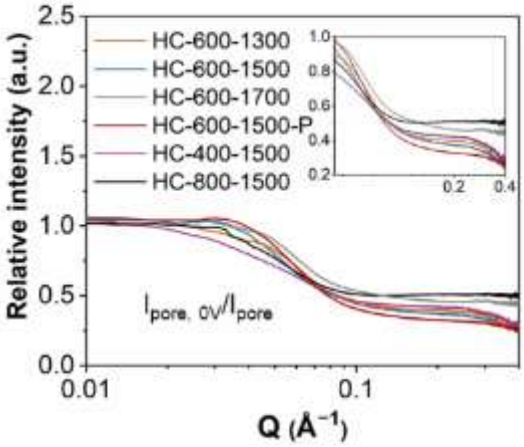

Figure S1.3 Relative SAXS profiles of fully sodiated HC versus pristine electrodes.

The scattering intensity of the nanopores was uniformly decreasing in the range from 0.08 to 0.4 Å^{-1} , indicating that the pores were uniformly filled. We fitted the SAXS curves of fully sodiated HC using the uniform model of filled pores.

Uniform model of filled pores: In this model, we consider that all the pores are filled uniformly, at the same volume percent, and thus we have a constant electron density for all the pores[6].

$$I_{mp}(Q)=\text{scale}(\Delta\text{SLD}_{\text{HC}}-\Delta\text{SLD}_{\text{pore}})^2\int_0^\infty |F(Q,R)|^2\,V^2(R)\,N\,P(R)\,dR \qquad \text{(S27)}$$

2 Supporting Tables

Table S1 The specific surface area and pore structure parameters measured by N_2 adsorption/desorption of LSC-400, LSC-600, and LSC-800.

**Sample**	**SSA[a] (m^2 g^{-1})**	**V_{total} (cm^3 g^{-1})**	**V_{meso} (cm^3 g^{-1})**	**V_{micro} (cm^3 g^{-1})**	**Average pore (nm)**
LSC-400	2.6	0.00598	0.00515	0.000833	/
LSC-600	201.0	0.116	0.0233	0.0927	2.19
LSC-800	438.6	0.193	0.0370	0.156	1.79

a: BET specific surface area

Table S2 The specific surface area and pore structure parameters measured by N_2 adsorption/desorption of HC-400-1500, HC-800-1500, HC-600-1300, HC-600-1500, HC-600-1700, and HC-600-1500-P.

Sample	Pore structure parameters			
	**SSA[a]** ($m^2\ g^{-1}$)	**V_{total}** ($cm^3\ g^{-1}$)	**V_{meso}** ($cm^3\ g^{-1}$)	**V_{micro}** ($cm^3\ g^{-1}$)
HC-400-1500	6.1	0.015	0.015	not detected
HC-800-1500	123.8	0.074	0.045	0.029
HC-600-1300	19.7	0.017	0.0092	0.0078
HC-600-1500	8.1	0.021	0.02048	0.00052
HC-600-1700	4.6	0.013	0.01263	0.00037
HC-600-1500-P	3.7	0.0068	0.00639	0.00041

a: BET specific surface area

Table S3 The specific surface area and pore structure parameters measured by CO_2 adsorption/desorption of HC-400-1500, HC-800-1500, HC-600-1300, HC-600-1500, HC-600-1700, and HC-600-1500-P.

**Sample**	**Pore structure parameters**	
	**SSA**[a] ($m^2\ g^{-1}$)	$\mathbf{V_{pore\ size\ <\ 0.7\ nm}}$ ($cm^3\ g^{-1}$)
HC-400-1500	40.5	0.0024
HC-800-1500	236.6	0.041
HC-600-1300	62.9	0.014
HC-600-1500	37.4	0.0026
HC-600-1700	18.3	0.0011
HC-600-1500-P	14.4	0.00094

a: BET specific surface area

Table S4 Elemental content of LSC400, LSC600, and LSC800.

**Sample**	**Composition (at.%)**			
	C	H	S	O
LSC400	59.96	33.57	0.40	6.07
LSC600	75.91	20.31	1.09	2.69
LSC800	77.10	17.42	1.09	4.39

Table S5 Elemental content of HC-400-1500, HC-800-1500, HC-600-1300, HC-600-1500, HC-600-1700, and HC-600-1500-P.

**Sample**	**Composition (at.%)**		
	**C**	**H**	**O**
HC-400-1500	99.27	0.61	0.12
HC-800-1500	97.26	2.34	0.41
HC-600-1300	98.66	0.88	0.46
HC-600-1500	99.06	0.85	0.09
HC-600-1700	99.84	0.12	0.04
HC-600-1500-P	99.77	0.18	0.05

Note: LSC400 exhibited an elemental composition with 59.96 at.% carbon, 33.57 at.% hydrogen, 0.40 at.% sulfur, and 6.07 at.% oxygen, indicating a higher heteroatom proportion compared to LSC600 and LSC800 (Table S4). The HC-400-1500 sample displayed atomic percentages of 99.27 at.% carbon, 0.61 at.% hydrogen, and 0.12 at.% oxygen, closely resembling the composition of HC-600-1500 and HC-800-1500 (Table S5). Therefore, the nanopores in HC-400-1500 were formed through the elimination of heteroatoms and structural rearrangement of the carbon skeleton during the heat treatment process

Table S6 Morphological parameters of the nanopores deduced from SAXS patterns based on the Teubner-Strey model.

**Samples**	ρ_{struc} (g cm^{-3})	**ΔSLD** (*10^{-6} Å^{-2})	**d** (nm)	ξ (nm)	**D** (nm)	f_a	**Number of pores** **(10^{-3} nm^{-3})**	φ	**Pore volume** **(cm^3 g^{-1})**
HC-400-1500	2.26	19.2	5.28	0.43	1.86	0.58	99.5	33.5%	0.224
HC-800-1500	2.32	19.7	7.07	0.51	2.39	0.66	52.3	37.5%	0.259
HC-600-1300	2.22	18.9	5.54	0.51	1.97	0.50	75.7	30.3%	0.196
HC-600-1500	2.26	19.2	6.43	0.45	2.14	0.64	72.5	37.2%	0.262
HC-600-1700	2.30	19.6	7.97	0.48	2.49	0.74	51.3	41.4%	0.307
HC-600-1500-P	2.29	19.5	6.30	0.48	2.17	0.63	74.1	39.6%	0.286

Note:

ρ_{struc}: structural density

SLD: scattering length density

d: pore-pore distance

ξ: a correlation length that limits the extension of the order

D: pore diameter

φ: pore volume fraction

f_a: "amphiphilicity factor". The degree of connectivity of the nanopores in HC could be characterized by the "amphiphilicity factor" (f_a)[4]. A higher f_a value indicates higher disorganization and deviation from a spherical shape of the nanopores in the HC, as well as increased pore connectivity.

Number of pores: The number of pores is equal to the volume of the pores divided by the volume of the individual pores (the volume of the individual pores calculated from the average pore diameter).

$$Number\ of\ pores = \frac{Pore\ volume}{\frac{4}{3}\pi\left(\frac{D}{2}\right)^3} = \frac{6 \cdot Pore\ volume}{\pi D^3} \qquad (S28)$$

Table S7 The pore size distribution parameters of HC nanopores modeled by Schulz-Zimm particle size distribution

Samples	Average pore diameters (nm)	FWHM pore size distribution (Å)
HC-400-1500	1.96	5.81
HC-800-1500	2.44	6.34
HC-600-1300	1.92	6.69
HC-600-1500	2.10	6.20
HC-600-1700	2.44	5.86
HC-600-1500-P	2.16	6.23

Table S8 Crystalline structure parameters measured by XRD for HC-400-1500, HC-800-1500, HC-600-1300, HC-600-1500, HC-600-1700, and HC-600-1500-P.

Samples	d_{002} (nm)	L_c (nm)	N	d_{100} (nm)	L_a (nm)
HC-400-1500	0.380	1.15	4.03	0.205	4.38
HC-800-1500	0.371	1.41	4.80	0.205	5.30
HC-600-1300	0.391	0.86	3.23	0.204	4.09
HC-600-1500	0.381	1.17	4.05	0.205	4.81
HC-600-1700	0.368	1.95	6.34	0.207	6.51
HC-600-1500-P	0.383	1.18	4.02	0.203	4.82

Table S9 The A_{Dx}/A_G value calculated by Raman spectra for HC-400-1500, HC-800-1500, HC-600-1300, HC-600-1500, HC-600-1700, and HC-600-1500-P.

**Sample**	**A_D / A_G**	**A_{D1} / A_G**	**A_{D2} / A_G**
HC-600-1300	1.49	0.49	0.85
HC-600-1500	1.40	0.35	0.65
HC-600-1700	1.32	0.30	0.47
HC-600-1500-P	1.35	0.34	0.65
HC-400-1500	1.37	0.45	0.66
HC-800-1500	1.39	0.37	0.66

Table S10 △SLD and SLD of the raw HC electrode and the HC electrode at 0 V and the pore-filling ratios of HC samples.

**Samples**	**ΔSLD_{HC}[a] (*10^{-6} Å^{-2})**	**$\Delta SLD_{HC, 0V}$[b] (*10^{-6} Å^{-2})**	**$(\Delta SLD_{HC, 0V}/\Delta SLD_{HC})^2$**	**$SLD_{pore, 0V}$[c] (*10^{-6} Å^{-2})**	**$\rho_{pore, 0V}$[d] (g cm^{-3})**	**Pore-filling ratio[e]**
HC-400-1500	19.2	12.03	0.393	7.17	0.872	90.1%
HC-800-1500	19.7	14.55	0.529	5.45	0.663	68.5%
HC-600-1300	18.9	11.71	0.384	7.19	0.875	90.4%
HC-600-1500	19.2	11.88	0.383	7.32	0.891	92.0%
HC-600-1700	19.6	14.31	0.533	5.29	0.644	66.5%
HC-600-1500-P	19.5	12.12	0.382	7.38	0.898	92.7%

a: The SLD of the raw electrode is 0 Å^{-2}. $\Delta SLD_{HC} = SLD_{HC} - SLD_{pore} = SLD_{HC}$

b: $\Delta SLD_{HC, 0V} = SLD_{HC} - SLD_{pore, 0V}$, $SLD_{pore, 0V} \neq 0$

c: The $SLD_{pore, 0V}$ is the SLD of the nanopore of fully sodiated HC.

d: $\rho_{pore, 0V}$ is the density of the pores after filling with sodium clusters.

e: When the pores are completely filled with sodium clusters, the density of the pores is 0.968 g cm^{-3}.Therefore, the pore-filling ratio is equal to the pore density divided by the sodium cluster density (0.968 g cm^{-3}).

Table S11 Reduced chi values calculated from experimental and fitted curves of SAXS for HC-400-1500, HC-800-1500, HC-600-1300, HC-600-1500, HC-600-1700, and HC-600-1500-P.

Samples	Reduced chi %
HC-400-1500	1.22
HC-800-1500	2.39
HC-600-1300	2.53
HC-600-1500	1.15
HC-600-1700	3.63
HC-600-1500-P	2.68

Note: The fit degree of the SAXS curve was confirmed by the chi-square check. A chi-square value <5% indicates that the fitted curve has a favorable degree of fit. The chi-square and reduced chi value are calculated as shown in the following equation:

$$\chi^2=\sum_l^N\left(\frac{data_i\text{-}model_i}{model_i}\right)^2/(N-N_{varys}) \qquad \text{(S29)}$$

$$\chi_{reduced}=\sqrt{\chi^2} \qquad \text{(S30)}$$

Where χ^2 is the chi-squared, $data_i$ are experimental datas, $model_i$ are model fit datas, $\chi_{reduced}$ is the reduced chi, N is the number of data points, and N_{varys} is the number of variables in the fitting process.

Table S12 Reduced chi values calculated from experimental and fitted curves of SAXS for HC electrodes at 0 V.

**Samples**	**Reduced chi %**
HC-400-1500-0V	3.11
HC-800-1500-0V	3.54
HC-600-1300-0V	3.41
HC-600-1500-0V	4.14
HC-600-1700-0V	3.32
HC-600-1500-P-0V	3.59

Table S13 Internal resistance (R_s), SEI films resistance (R_{SEI}), charge transfer resistance (R_{ct}) of HC-400-1500, HC-800-1500, HC-600-1300, HC-600-1500, HC-600-1700, and HC-600-1500-P

**Samples**	**R_s (Ω)**	**R_{SEI} (Ω)**	**R_{ct} (Ω)**
HC-400-1500	3.21	1.56	40.6
HC-800-1500	4.61	3.04	126.3
HC-600-1300	2.49	1.87	45.6
HC-600-1500	4.29	1.79	46.2
HC-600-1700	3.85	1.58	139.3
HC-600-1500-P	5.16	1.43	48.4

Table S14 True density measured by helium pycnometry, pore volume fraction, and pore volume calculated by true density

**Samples**	ρ_{He} [a] (g cm^{-3})	**Pore volume fraction** φ	**Pore volume** (cm^3 g^{-1})
HC-400-1500	1.631	27.8%	0.171
HC-800-1500	1.996	14.0%	0.070
HC-600-1300	2.120	4.5%	0.021
HC-600-1500	1.457	35.5%	0.244
HC-600-1700	1.357	41.0%	0.302
HC-600-1500-P	1.413	38.3%	0.271
Graphite	2.258	/	/

a: $\boldsymbol{\rho_{He}}$ is the density of HC measured by helium pycnometry.

Note: The relationship between structural density ρ_{struc}, pore density ρ_{pore}, and true density ρ_{He} of HC is as follows:

$$(1-\varphi)\cdot\rho_{struc}+\varphi\cdot\rho_{pore}=\rho_{He} \qquad \text{(S31)}$$

where ρ_{pore} is 0, resulting in a pore volume fraction φ as:

$$\varphi=1-\frac{\rho_{He}}{\rho_{struc}} \qquad \text{(S32)}$$

The pore volume V_{pore} could be calculated as by the following equation:

$$V_{pore}=\frac{1}{\rho_{He}}-\frac{1}{\rho_{struc}}=\frac{\varphi}{\rho_{He}} \qquad \text{(S33)}$$

Table S15 Pore volume fraction, density, and pore volume of HC calculated by SAXS

**Samples**	**Pore volume fraction** φ	ρ_{sample} **(g cm^{-3})**	**Pore volume (cm^3 g^{-1})**
HC-400-1500	33.5%	1.503	0.224
HC-800-1500	37.5%	1.450	0.259
HC-600-1300	30.3%	1.547	0.196
HC-600-1500	37.2%	1.419	0.262
HC-600-1700	41.4%	1.348	0.307
HC-600-1500-P	39.6%	1.383	0.286

Note: The ρ_{sample} of HC is calculated from the following equation:

$$\rho_{sample}=(1-\varphi)\rho_{struc} \qquad \text{(S34)}$$

The pore volume is calculated according to Equation S33.

Table S16 True densities from butanol pycnometry measurements, pore volume fractions and pore volumes calculated by true density

**Samples**	**ρ_{buta} [a] (g cm^{-3})**	**Pore volume fraction φ**	**Pore volume (cm^3 g^{-1})**
HC-400-1500	1.495	33.9%	0.226
HC-800-1500	1.496	35.5%	0.237
HC-600-1300	1.539	30.6%	0.199
HC-600-1500	1.416	37.3%	0.264
HC-600-1700	1.353	41.2%	0.304
HC-600-1500-P	1.381	39.7%	0.287
Graphite	2.247	/	/
YP50F	2.199	0.50%	0.002

a: ρ_{buta} is the density of HC measured by butanol pycnometry.

Note: The pore volume fraction and pore volume are calculated according to Equations S32 and S33.

Table S17 The volume of nanopore accessible to N_2, nanopore inaccessible to N_2 but accessible to CO_2, nanopore accessible to helium, nanopore accessible to the electrolyte

Sample	Volume of nanopore (cm^3 g^{-1})			
	accessible to N_2 [a]	inaccessible to N_2 but accessible to CO_2 [b]	accessible to helium [c]	accessible to the electrolyte [d]
HC-600-1300	0.0078	0.014	0.178	0
HC-600-1500	0.00052	0.0026	0.019	0
HC-600-1700	0.00037	0.0011	0.002	0.003
HC-600-1500-P	0.00041	0.00094	0.016	0
HC-400-1500	0	0.0024	0.055	0
HC-800-1500	0.027	0.041	0.167	0.022

a: The volume of nanopore accessible to N_2 is the volume of micropores measured by N_2 adsorption/desorption.

b: The pore distributions of HC-400-1500, HC-800-1500, HC-600-1300, HC-600-1500, HC-600-1700, and HC-600-1500-P were identified to be above 0.9 nm by nitrogen adsorption/desorption (Figure S3 and S6). While the pore distributions of HC-400-1500, HC-800-1500, HC-600-1300, HC-600-1500, HC-600-1700, and HC-600-1500-P were shown to be lower than 0.9 nm from the CO_2 adsorption/desorption measurements (Figures S2 and 3g). Therefore, the volume of nanopore inaccessible to N_2 but accessible to CO_2 was the volume of micropores obtained from CO_2 adsorption/desorption.

c: The volume of nanopore accessible to helium is the pore volume obtained by butanol pycnometry minus the pore volume obtained by helium pycnometry.

d: The volume of nanopore accessible to the electrolyte is approximately equal to the pore volume calculated by SAXS minus the pore volume obtained by butanol pycnometry. When the minus value is negative, the volume of nanopore accessible to the electrolyte could be identified as 0 cm^3 g^{-1}.

Table S18 Crystalline structure parameters measured by XRD.

**Samples**	**d_{002} (nm)**	**L_c (nm)**	**N**	**d_{100} (nm)**	**L_a (nm)**	**ρ_{struct} (g cm^{-3})**
B-HC-1300	0.383	0.89	3.26	0.200	4.60	2.22
B-HC-1500	0.376	1.04	3.72	0.201	4.79	2.24
C-HC-1300	0.380	1.00	3.60	0.199	3.94	2.26
C-HC-1500	0.378	1.11	3.90	0.199	4.36	2.27
AL-HC-1300	0.382	0.90	3.31	0.199	5.07	2.25
AL-HC-1500	0.372	1.05	3.75	0.201	5.20	2.26
EHL-HC-1300	0.377	1.14	3.97	0.200	5.24	2.26
EHL-HC-1500	0.369	1.24	4.30	0.202	5.96	2.26
G-HC-1300	0.385	1.04	3.69	0.199	5.14	2.23
G-HC-1500	0.364	1.24	4.35	0.203	6.47	2.27
HG-HC-1300	0.393	0.95	3.40	0.199	4.92	2.19
HG-HC-1500	0.380	1.07	3.82	0.201	5.09	2.22

Table S19 True densities obatianed from butanol pycnometry, pore volume fractions, and pore volumes calculated by true density, plateau-potential capacity, volume fraction of sodium filling, and pore-filling ratios of HCs.

**Samples**	ρ_{buta} (g cm^{-3})	**Pore volume fraction** φ	**Pore volume** (cm^3 g^{-1})	**Plateau-potential capacity** (mAh g^{-1})	**Volume fraction of sodium filling**[a] (φ_{Na})	**Pore-filling ratios**
B-HC-1300	1.606	27.7%	0.172	184	26.1%	94.5%
B-HC-1500	1.557	30.5%	0.196	208	28.7%	94.2%
C-HC-1300	1.511	33.2%	0.220	228	30.4%	91.8%
C-HC-1500	1.496	34.1%	0.228	229	30.4%	89.0%
AL-HC-1300	1.581	29.7%	0.188	200	28.1%	94.4%
AL-HC-1500	1.552	31.3%	0.202	216	29.7%	94.7%
EHL-HC-1300	1.556	31.2%	0.200	208	30.0%	96.5%
EHL-HC-1500	1.521	32.7%	0.215	207	27.9%	85.3%
G-HC-1300	1.670	25.1%	0.150	144	21.3%	84.8%
G-HC-1500	1.610	29.1%	0.181	164	23.3%	80.2%
HG-HC-1300	1.504	31.3%	0.208	224	29.8%	95.1%
HG-HC-1500	1.499	32.5%	0.217	218	28.9%	89.0%

Note: The pore volume fraction and pore volume are calculated according to Equations S32 and S33.

a: The volume fraction of sodium filling (φ_{Na}) is calculated by the formula

$$\varphi_{Na} = \frac{C_{plat} \times \rho_{butanol}}{C_{v,Na}} \qquad \text{(S35)}$$

where C_{plat} is the plateau-potential capacity, ρ_{buta} is the density of HC obtained from butanol pycnometry, $C_{v,Na}$ is the theoretical pore volumetric specific capacity (1130 mAh cm^{-3}). $\frac{C_{plat}}{C_{v,\,Na}}$ is

the volume of the sodium cluster. $\frac{1}{\rho_{buta}}$ is the HC volume. The volume fraction of sodium filling is the volume of sodium clusters divided by the HC volume.

Table S20 Morphological parameters of the nanopores deduced from SAXS patterns based on the Teubner-Strey model.

Sample	ρ_{struct} (g cm^{-3})	ΔSLD (*10^{-6} Å^{-2})	d (nm)	ξ (nm)	D (nm)	Micropore volume fraction φ	f_a	Pore-filling ratio %	Volume^{SAXS} (cm^3 g^{-1})
B-HC-1300	2.22	18.9	6.05	0.47	2.09	27.8%	0.49	93.9%	0.173
B-HC-1500	2.24	19.1	5.38	0.50	1.92	31.1%	0.62	92.3%	0.202
C-HC-1300	2.26	19.2	5.01	0.52	1.75	32.8%	0.40	92.7%	0.216
C-HC-1500	2.25	19.1	5.14	0.50	1.82	33.3%	0.45	91.3%	0.222
AL-HC-1300	2.25	19.1	5.21	0.48	1.85	29.8%	0.49	94.3%	0.189
AL-HC-1500	2.25	19.1	5.64	0.51	2.00	32.0%	0.51	92.8%	0.209
EHL-HC-1300	2.26	19.2	5.51	0.50	1.96	31.8%	0.52	94.3%	0.206
EHL-HC-1500	2.26	19.2	5.97	0.51	2.11	33.1%	0.55	84.3%	0.219
G-HC-1300	2.23	19.0	7.28	0.47	2.34	24.8%	0.71	85.9%	0.158
G-HC-1500	2.27	19.3	8.04	0.48	2.48	28.8%	0.75	80.9%	0.178
HG-HC-1300	2.19	19.1	4.80	0.49	1.68	31.8%	0.42	93.7%	0.213
HG-HC-1500	2.22	18.8	5.93	0.51	2.10	33.6%	0.55	86.0%	0.228

Note: EHL-HC-1500, G-HC-1300 and G-HC-1500 exhibited lower pore-filling ratios than other HC (Table S19 and S20). The low pore-filling ratio is attributed to the fact that the EHL-HC-1500 and G-HC-1500 possess small d_{002} values (EHL-HC-1500: 0.369 nm, G-HC-1500: 0.364 nm), long L_a (EHL-HC-1500: 5.96 nm, G-HC-1500: 6.47 nm), and excessive pore size

(EHL-HC-1500: 2.11 nm, G-HC-1500: 2.48 nm), and the G-HC-1300 exhibits a large pore diameter of 2.34nm (**Figure S25** and Table S18).

3 Supporting Figures

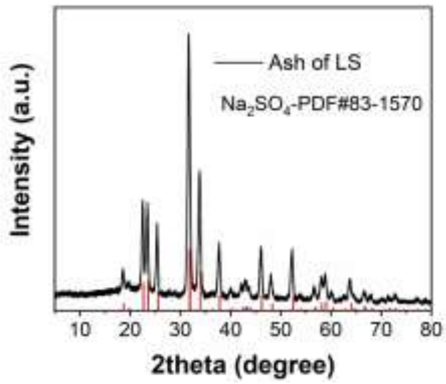

Figure S1 XRD patterns of products obtained by burning LS at 600°C for 2h in air atmosphere.

Note: The 0.5012 g of LS was burned in air and the remaining sample mass was 0.0950 g. The burning product of LS was analyzed by XRD as Na_2SO_4. The content of sulfonate groups in LS was calculated to be 2.67 mmol g^{-1} by material constancy of Na^+.

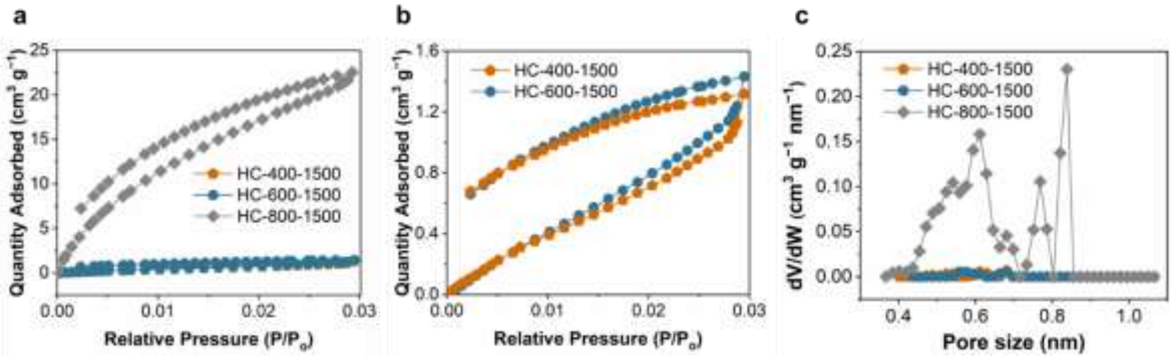

Figure S2. (a, b) CO_2 adsorption/desorption isotherms, (c) pore size distribution measured by CO_2 absorption/desorption for HC-400-1500, HC-600-1500, and HC-800-1500.

Note: For ultra-micropore materials with pores smaller than 0.7 nm, the diffusion of nitrogen molecules at a temperature of 77 K is hindered[9, 10]. Consequently, detecting the ultra-micropore structure of HC through nitrogen adsorption/desorption remains challenging. Despite the similarity in molecular size between CO_2 and N_2 molecules (0.330 nm and 0.364 nm), the high measurement temperature of CO_2 adsorption/desorption (273 K) facilitates the entry of CO_2 molecules with adequate kinetic energy into ultra-micropores[9, 11]. The ultra-micropore structure of HC was characterized by the CO_2 adsorption/desorption method.

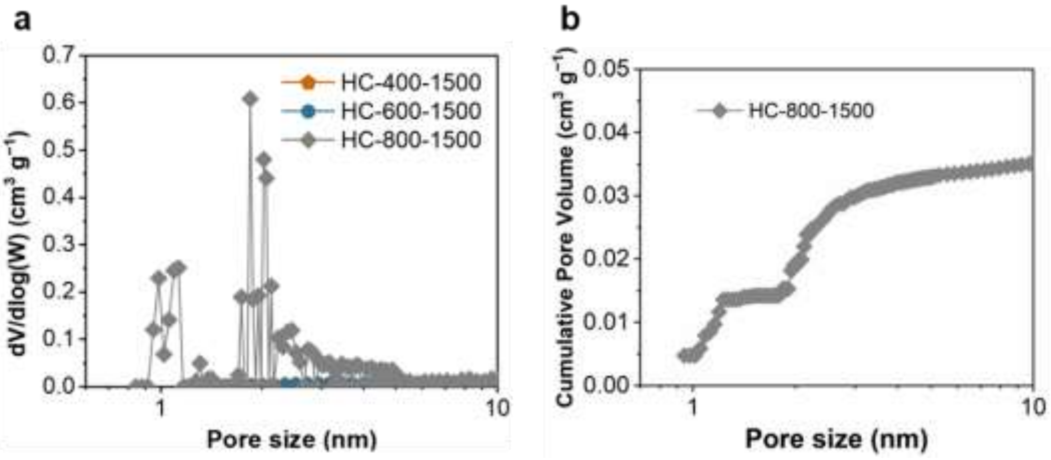

Figure S3. (a) Pore size distribution and (b) cumulative pore volume measured by N_2 absorption/desorption for HC-400-1500, HC-600-1500, and HC-800-1500.

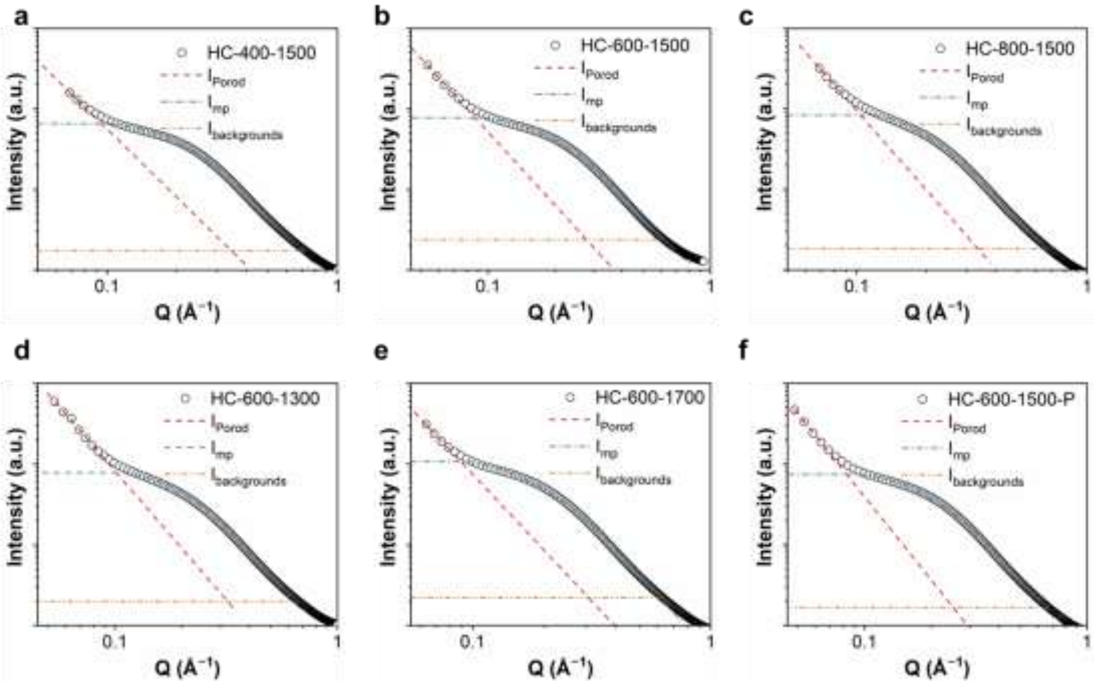

Figure S4. SAXS experimental and fitted curves of (a) HC-400-1500, (b) HC-600-1500, (c) HC-800-1500, (d) HC-600-1300, (e) HC-600-1700, and (f) HC-600-1500-P.S

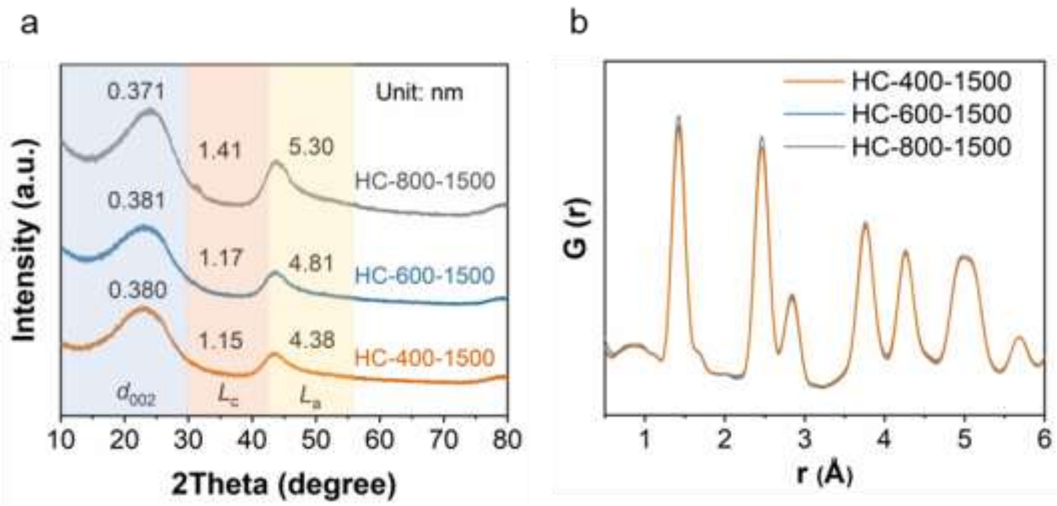

Figure S5. (a) XRD patterns and (b) PDF analysis obtained from neutron total scattering for HC-400-1500, HC-600-1500, and HC-800-1500.

Note: The value of d_1 corresponds to the bond length of the C-C bond (1.42 Å). The peak at d_3 = 2.84 Å represents the distance between C_1-C_4 carbon atoms in the benzene ring.

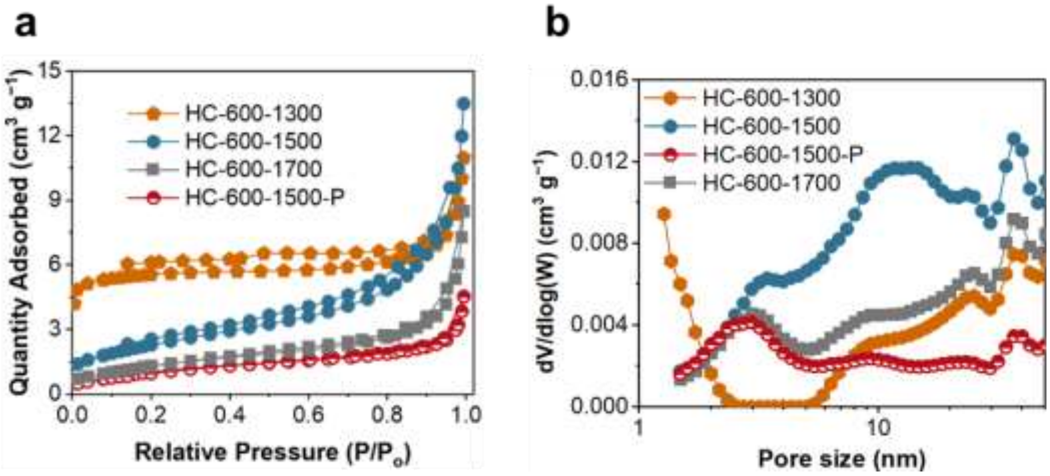

Figure S6. (a) N_2 adsorption/desorption isotherms, (b) pore size distribution for HC-400-1500, HC-600-1500, and HC-800-1500.

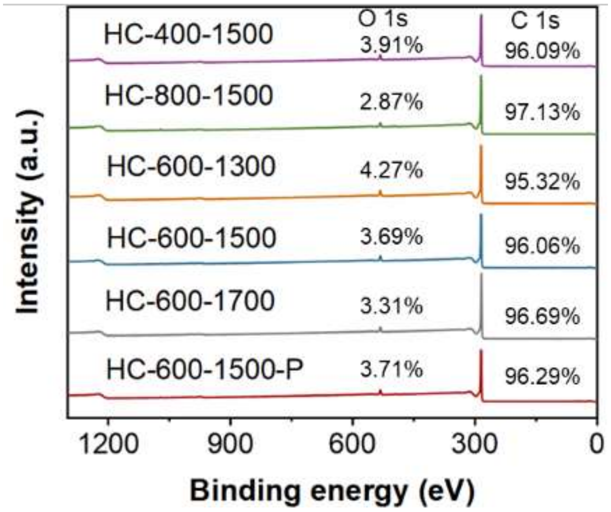

Figure S7. XPS survey spectra for HC-400-1500, HC-800-1500, HC-600-1300, HC-600-1500, HC-600-1700, and HC-600-1500-P.

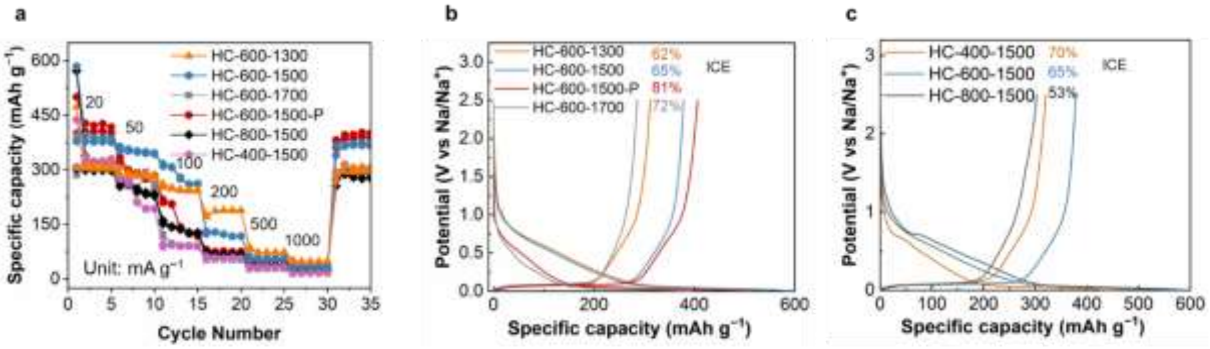

Figure S8. (a) Rate capability test and (b, c) initial GCD curves of HC-600-1300, HC-600-1500, HC-600-1700, HC-600-1500-P, HC-800-1500, and HC-400-1500 at a current density of 20 mA g^{-1}.

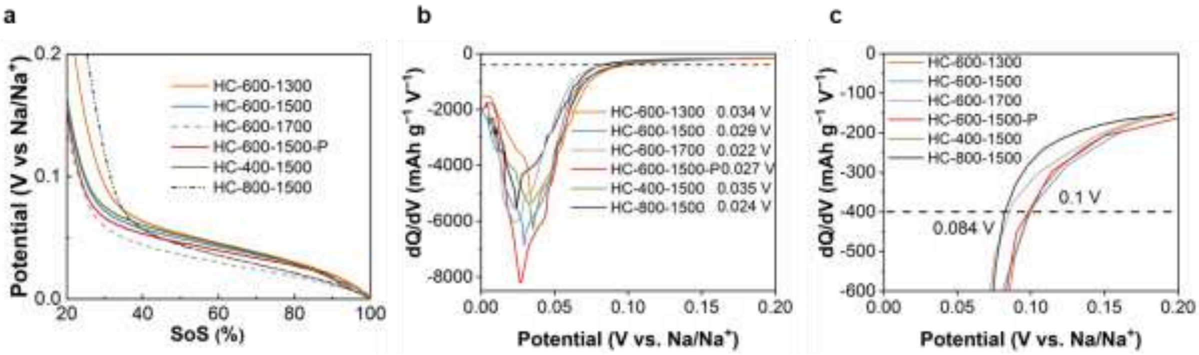

Figure S9. (a) Discharge curve, and (b，c) dQ/dV curve of HC-600-1300, HC-600-1500, HC-600-1700, HC-600-1500-P, HC-800-1500, and HC-400-1500 at a current density of 20 mA g^{-1}.

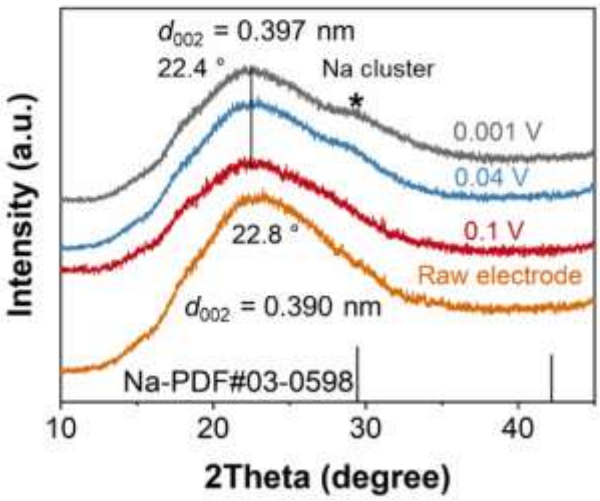

Figure S10. *Ex-situ* XRD pattern of HC-600-1300 electrodes.

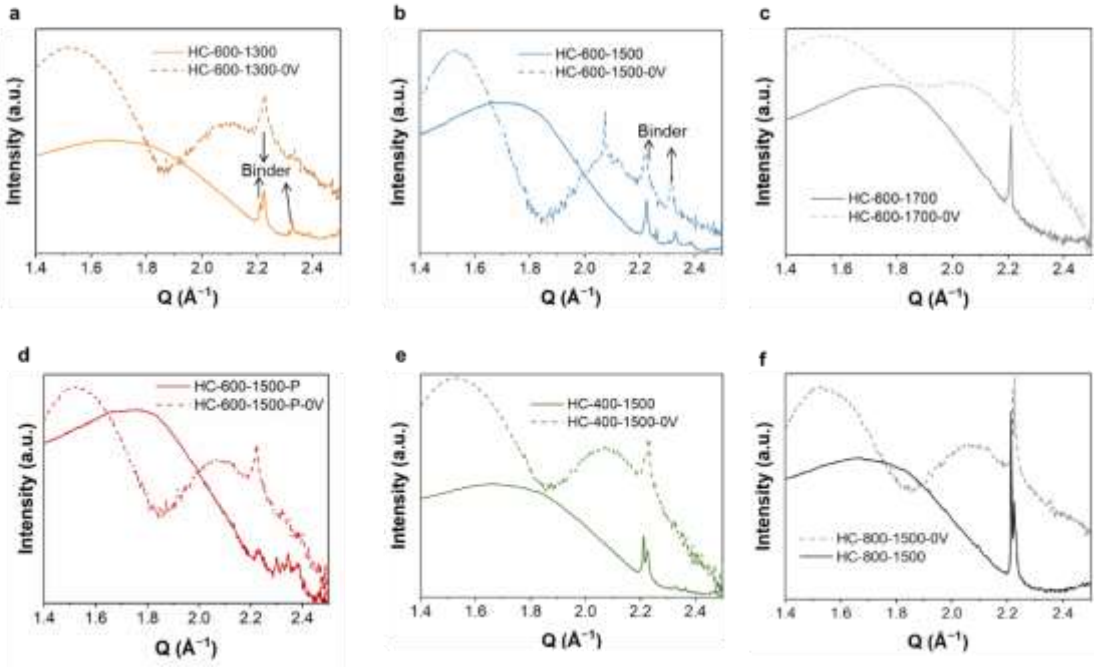

Figure S11. *Ex-situ* WAXS curves of (a) HC-600-1300, (b) HC-600-1500, (c) HC-600-1700, (d) HC-600-1500-P, (e) HC-400-1500, and (f) HC-800-1500.

Note: The weak X-ray intensity of laboratory XRD, coupled with the limitations of a 0-dimensional detector, often resulted in weak signals for the diffraction peaks of sodium clusters. To enhance the intensity and resolution of sodium cluster signal collection, we utilized WAXS with a synchrotron radiation source and a 2-dimensional detector.

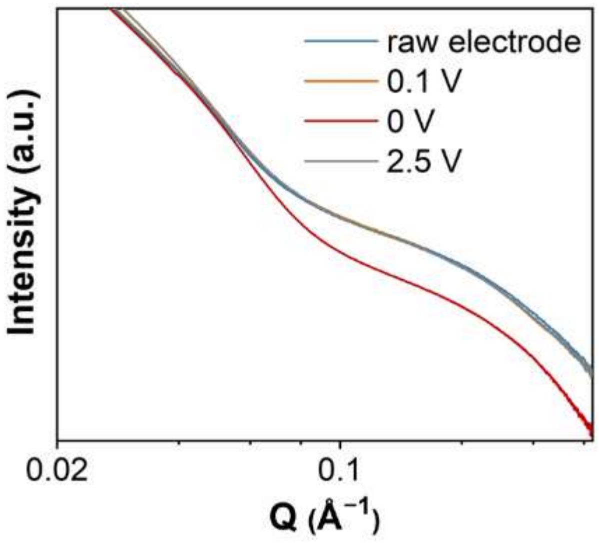

Figure S12. *Ex-situ* SAXS curves of HC-600-1300.

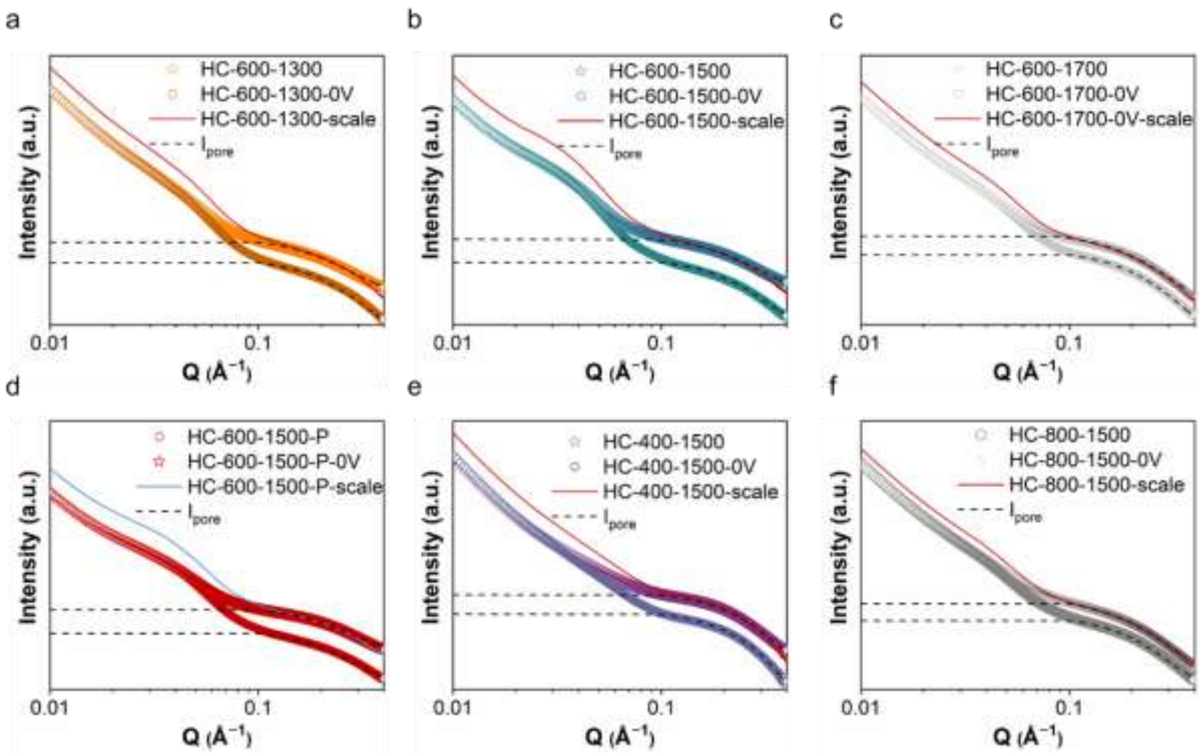

Figure S13. *Ex-situ* SAXS curves of (a) HC-600-1300, (b) HC-600-1500, (c) HC-600-1700, (d) HC-600-1500-P, (e) HC-400-1500, and (f) HC-800-1500. **Note:** The HC-X-Y-scale curve is the SAXS intensity of HC-600-1300-0V multiplied by $(\Delta SLD_{HC}/\Delta SLD_{HC,\,0V})^2$.

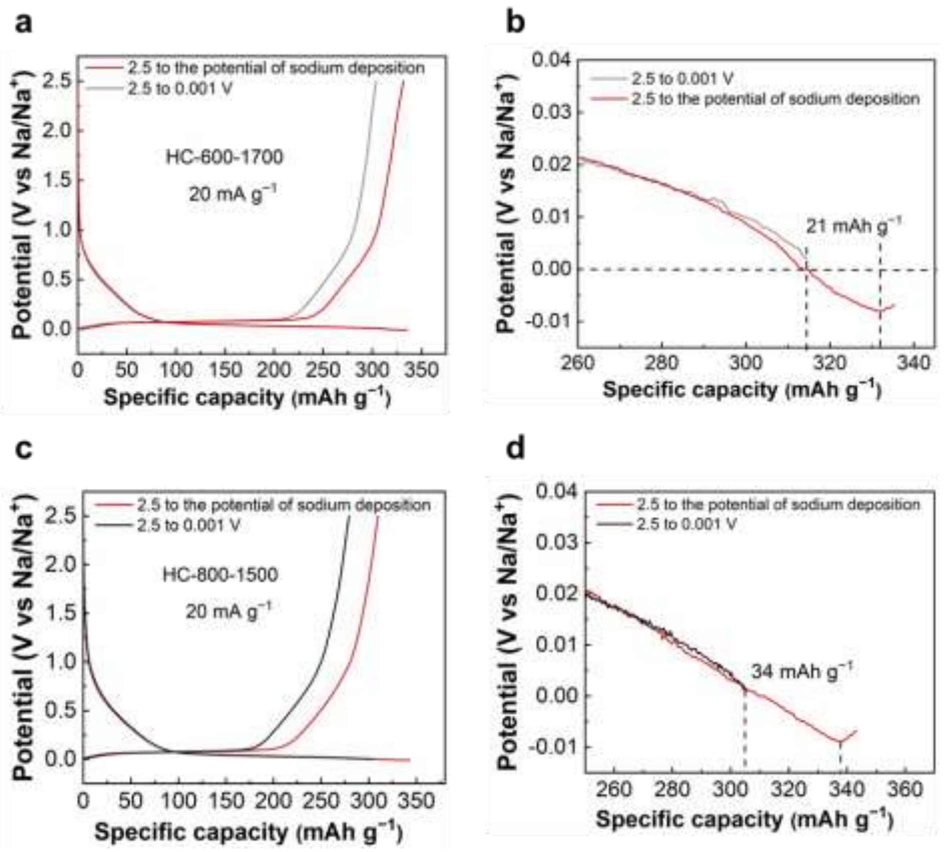

Figure S14. Galvanostatic discharge/charge curves for cut-off voltages of 0.001 V *vs.* Na/Na^+ and potential of sodium metal deposition at a current density of 20 mA g^{-1} for (a, b) HC-600-1700 and (c, d) HC-800-1700.

Note: The *ex-situ* ^{23}Na MAS ssNMR results indicate that nanopores with larger diameters results in the formation of larger sodium clusters (**Figure 3d**). The larger sodium clusters form with lower formation potentials (higher formation energy barrier)[12]. The filling of lithium in the closed nanopores also faces the same principle that larger pores need higher formation energy for Li clusters[13]. Moreover, from the previous theoretical simulations, it can also be concluded that the larger sodium clusters correspond to a lower potential for sodium cluster formation[13, 14].

From the normalized pore size distribution of HC samples at a sodiation potential of 0 V (**Figure 4c-d**), pores with diameters smaller than 40 Å can be filled with sodium clusters when discharged from 0.1 to 0 V *vs.* Na^+/Na, while pores with diameters larger than 40 Å are unable

to be filled. Therefore, we infer that the plateau-potential capacity of HC further discharged below 0 V *vs.* Na^+/Na before a potential inflection point (after which is the sodium plating on the surface of hard carbon) is attributed to the filling of sodium clusters in pores with diameters larger than 40 Å.

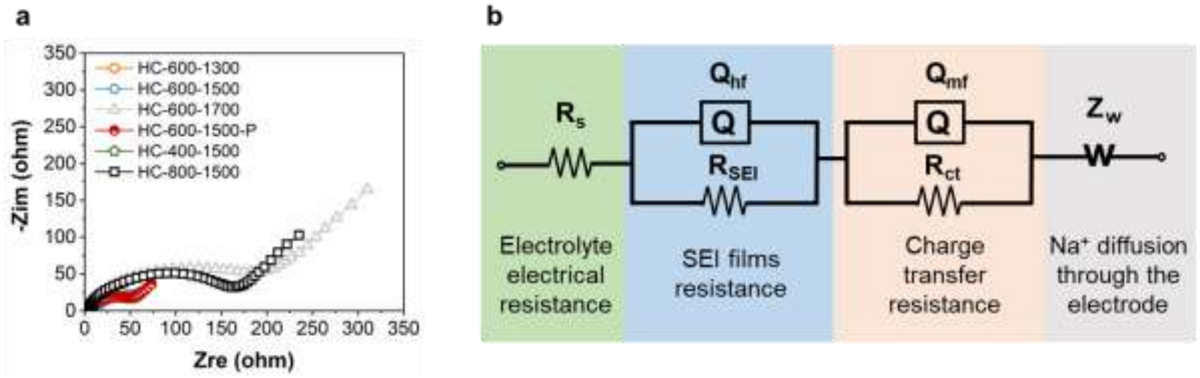

Figure S15. (a) Nyquist plots of HC-600-1300, HC-600-1500, HC-600-1700, HC-600-1500-P, HC-800-1500, and HC-400-1500. (b) An equivalent circuit was used for the fitting of the EIS data.

Note: In the plateau potential region of the discharge, the migration of Na^+ ion is generally divided into four processes: (1) migration of solvated Na^+ ions in the electrolyte; (2) desolvation of solvated Na^+ ions at the electrolyte-electrode interface; (3) diffusion of Na^+ ions through the solid-electrolyte interface (SEI)[15]; and (4) diffusion of Na^+ ions between interlayers and filling of Na^+ ions within the pores[16, 17]. The electrolyte electrical resistance (R_s) is related by the migration of solvated Na^+ ions in the electrolyte (1) and the resistance of the electrode.The SEI film resistance (R_{SEI}) is correlated with the desolvation process of solvated Na^+ ions (2) and the diffusion resistance of Na^+ ions in the SEI film (3). The charge transfer resistance (R_{ct}) is correlated with the diffusion resistance of Na^+ ions between interlayers and the energy barrier for Na^+ ions forming sodium clusters in the pores (4).

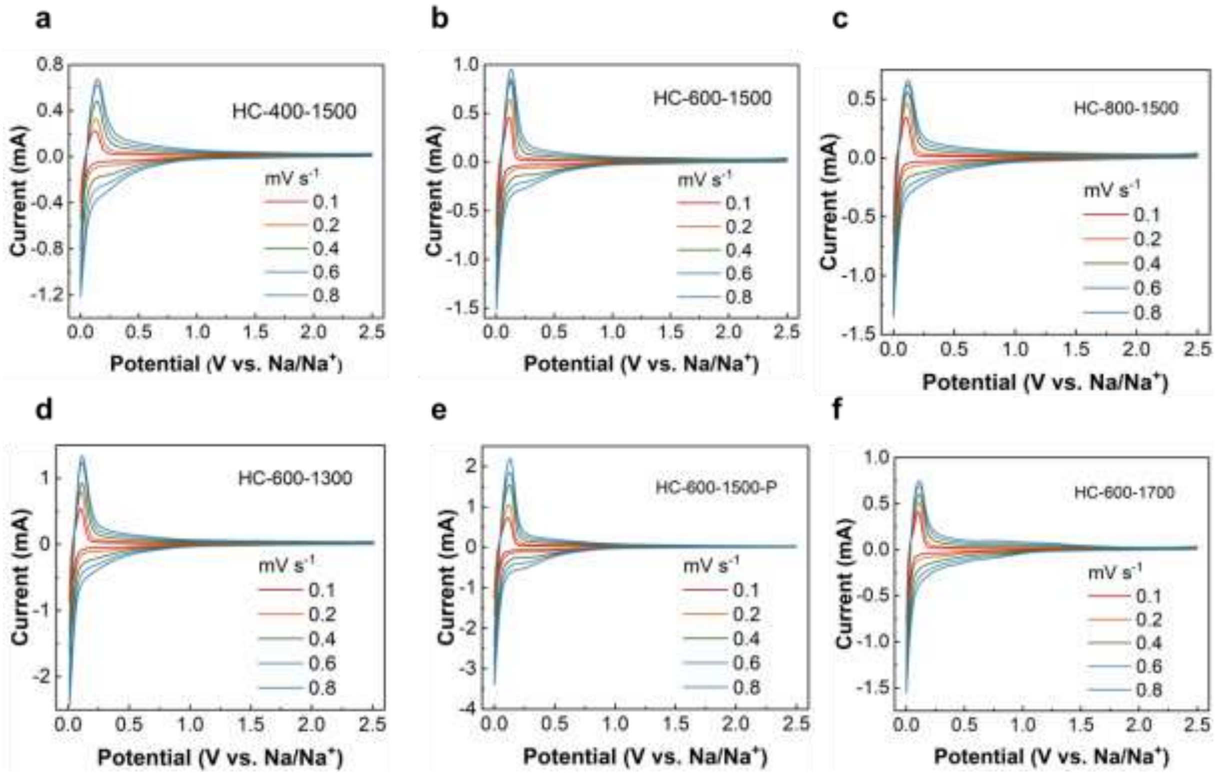

Figure S16. CV curves of (a) HC-400-1500, (b) HC-600-1500, (c) HC-800-1500, (d) HC-600-1300, (e) HC-600-1500-P, and (f) HC-600-1700.

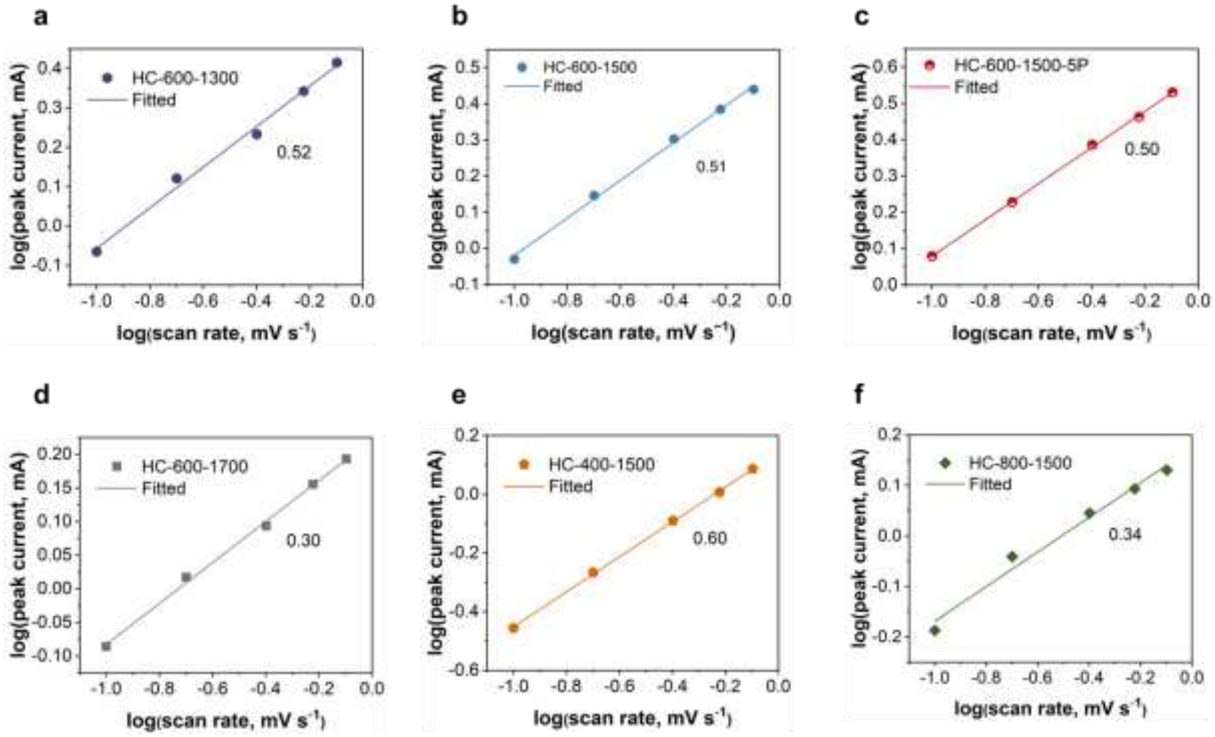

Figure S17. Linear relationship between log(peak current) and log(scan rate) of cathodic peaks for (a) HC-600-1300, (b) HC-600-1500, (c) HC-600-1500-P, (d) HC-600-1700, (e) HC-400-1500, and (f) HC-800-1500.

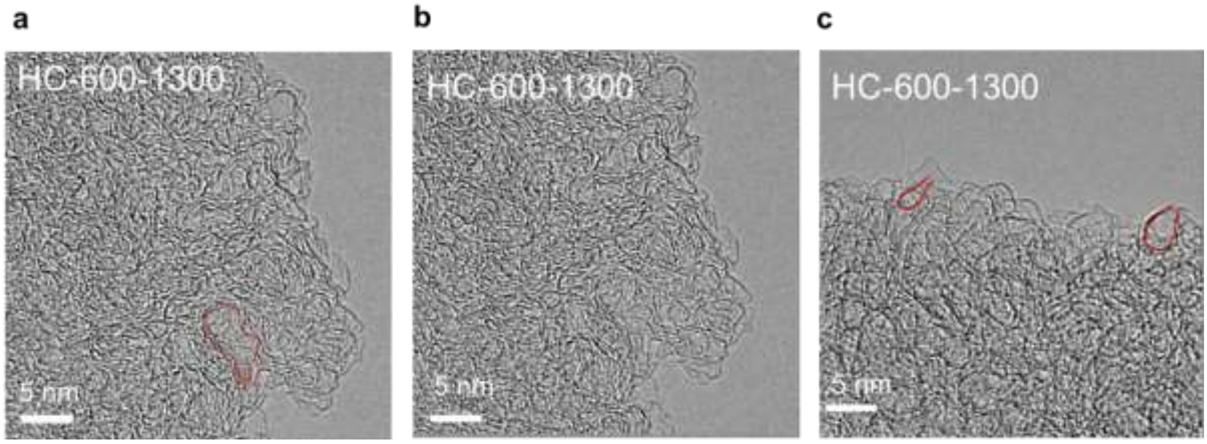

Figure S18. (a-c) HR-TEM images of HC-600-1300.

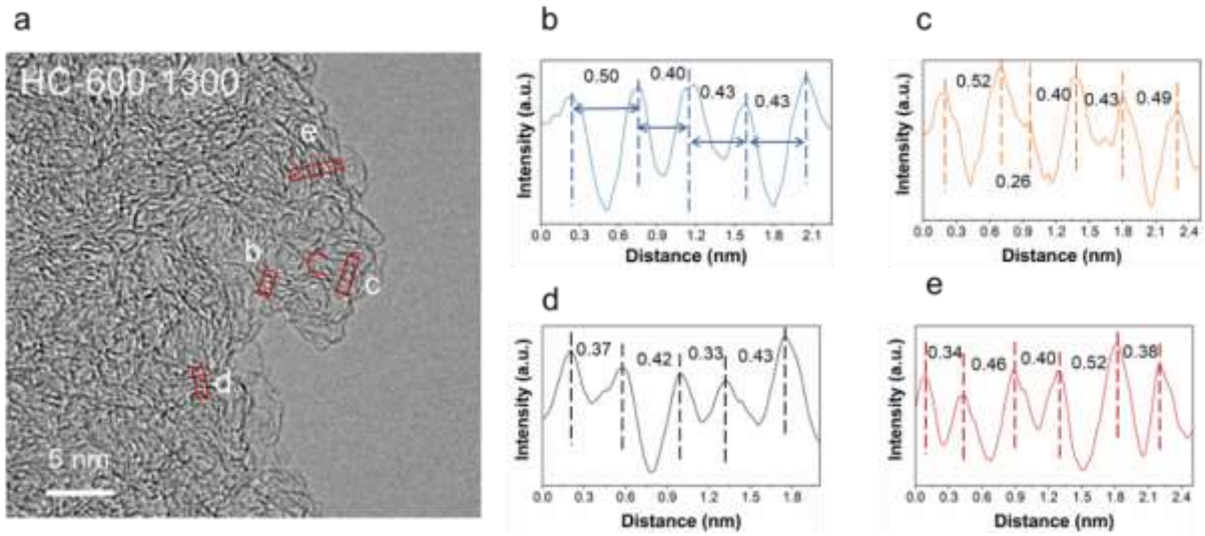

Figure S19. (a) HR-TEM image, and (b-e) the interlayer spacing in the corresponding region of HC-600-1300 in panel (a).

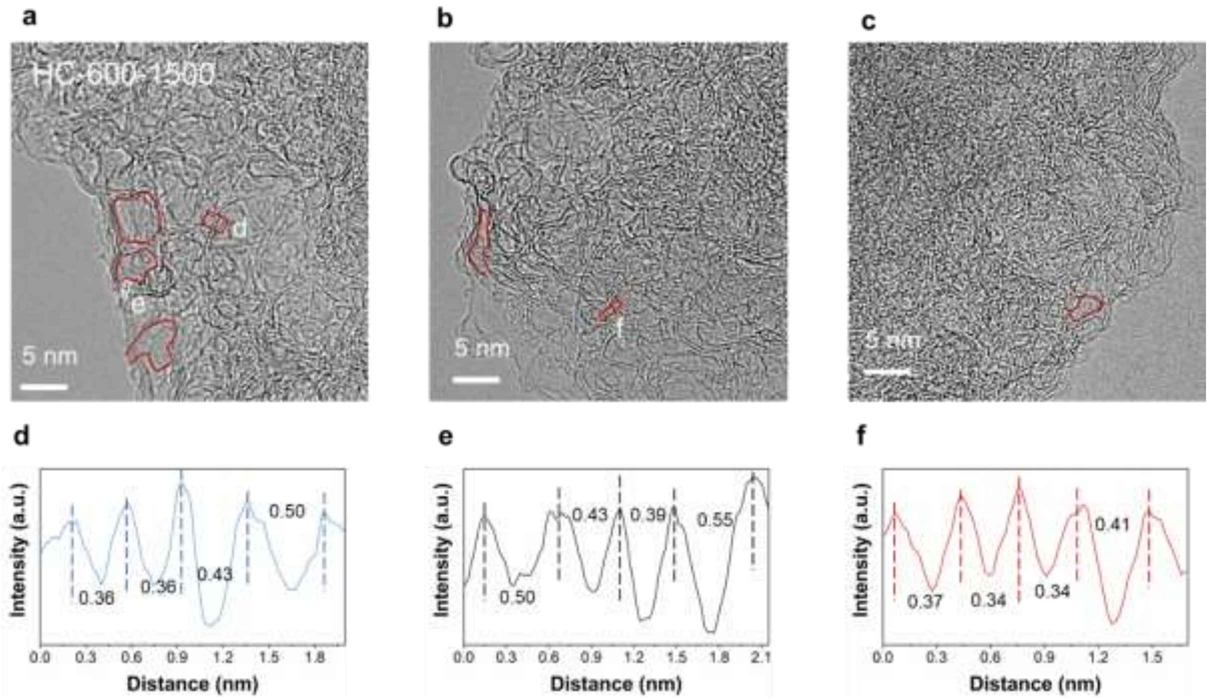

Figure S20. (a-c) HR-TEM image, and (d-f) the interlayer spacing in the corresponding region of HC-600-1500 in panel (a) and (b).

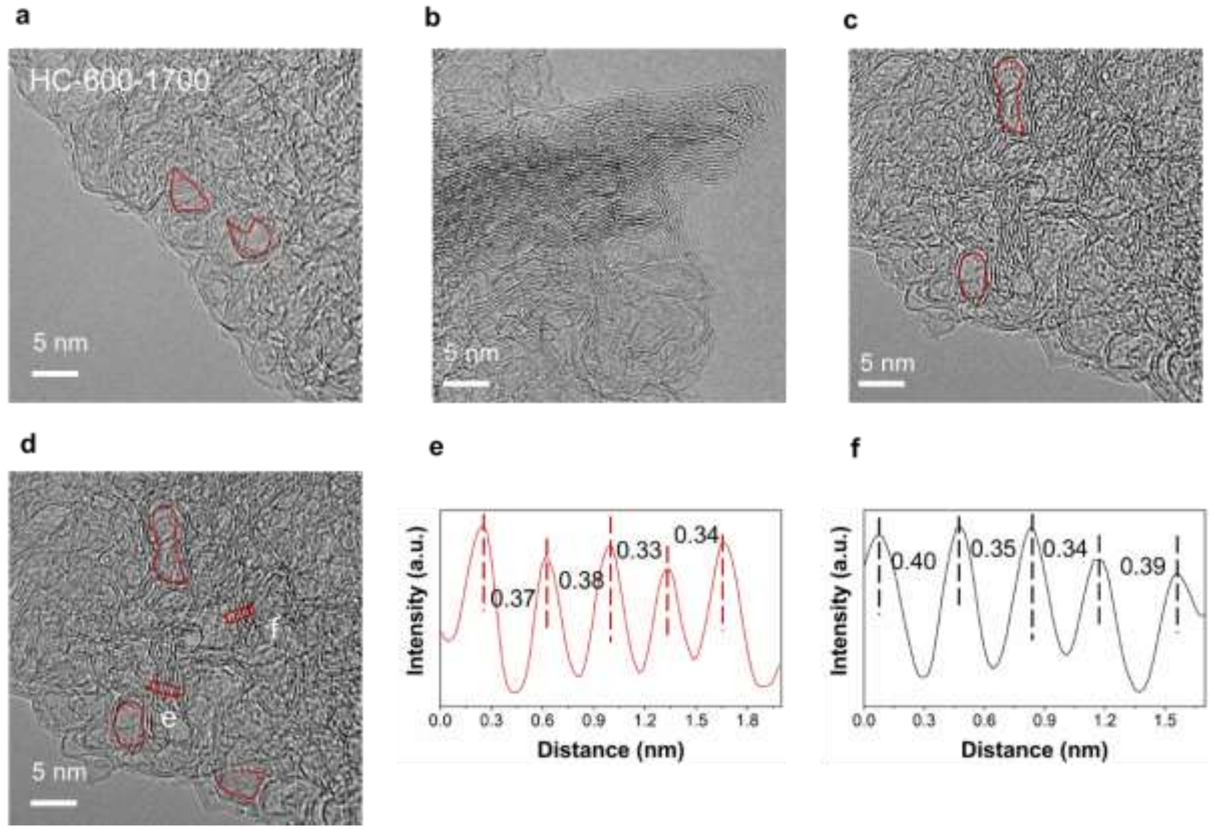

Figure S21. (a-d) HR-TEM image, and (e-f) the interlayer spacing in the corresponding region of HC-600-1700 in panel (d).

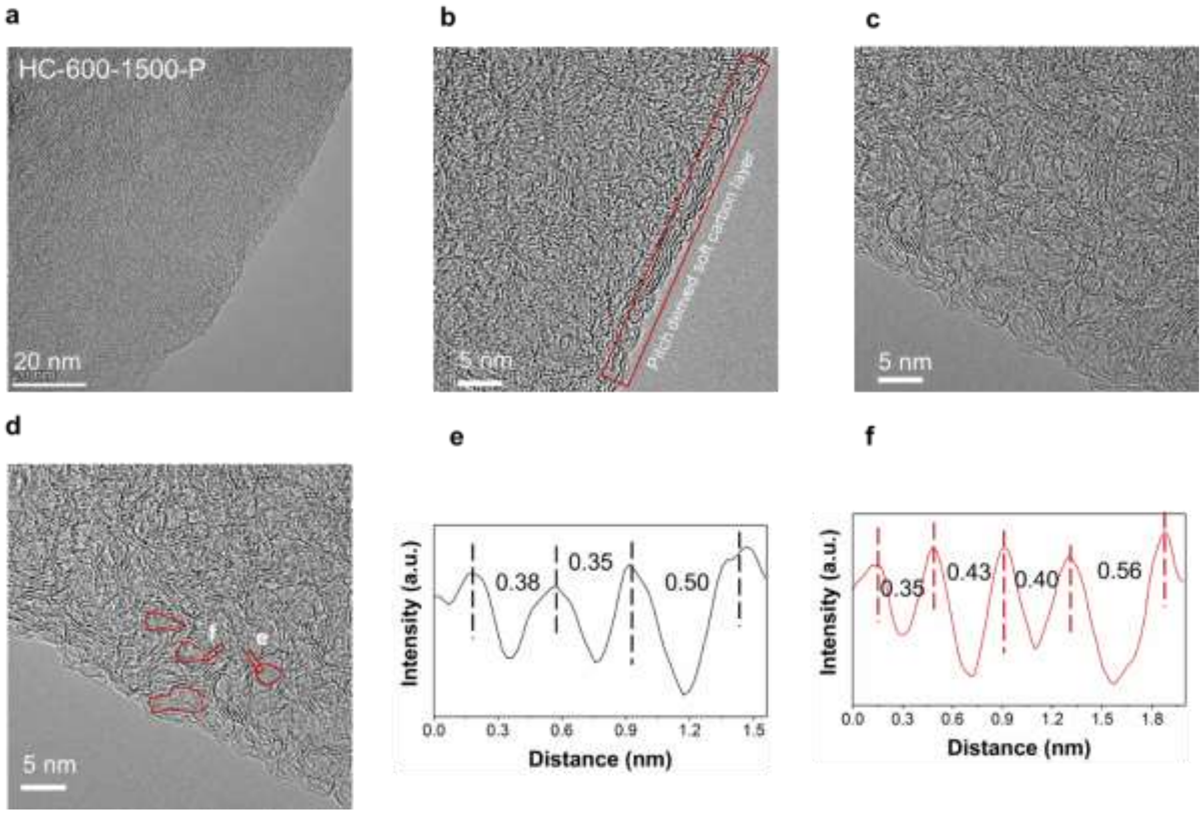

Figure S22. (a-d) HR-TEM image, and (e-f) the interlayer spacing in the corresponding region of HC-600-1500-P in panel (d).

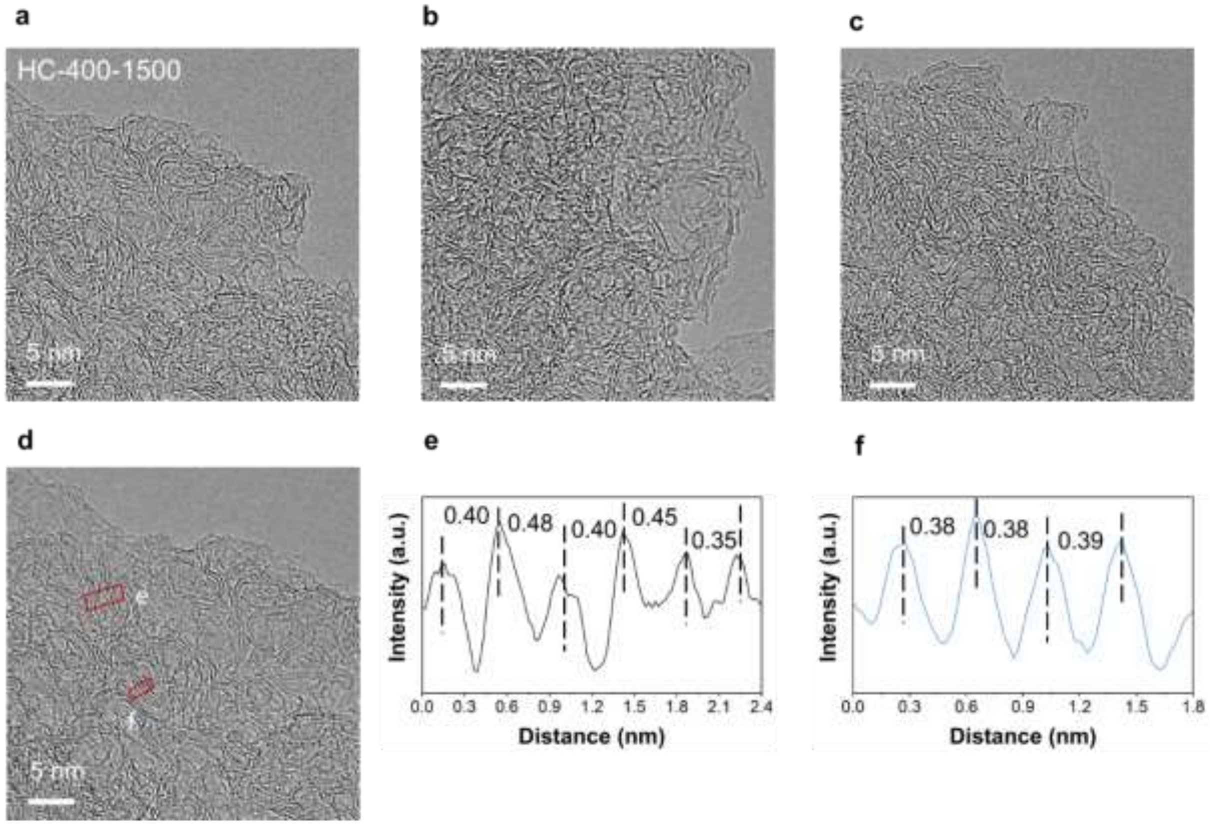

Figure S23. (a-d) HR-TEM image, and (e-f) the interlayer spacing in the corresponding region of HC-400-1500 in panel (d).

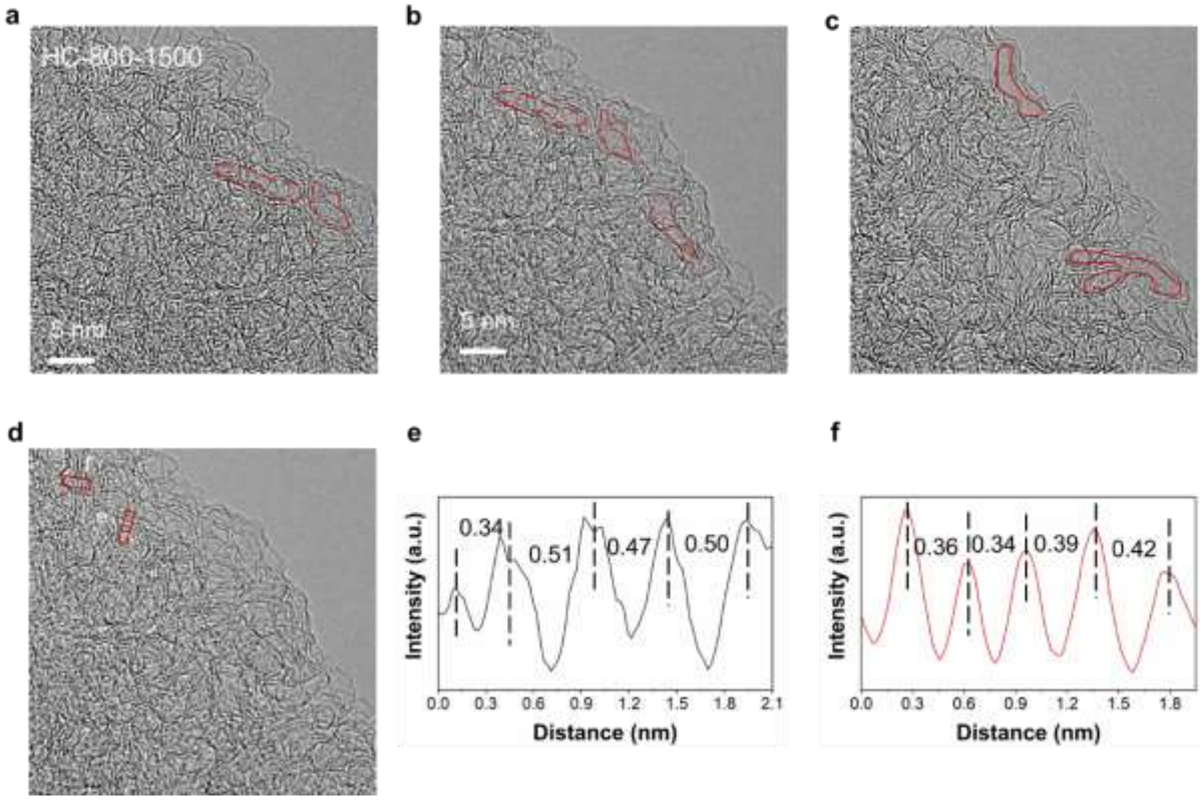

Figure S24. (a-d) HR-TEM image, and (e-f) the interlayer spacing in the corresponding region of HC-800-1500 in panel (d).

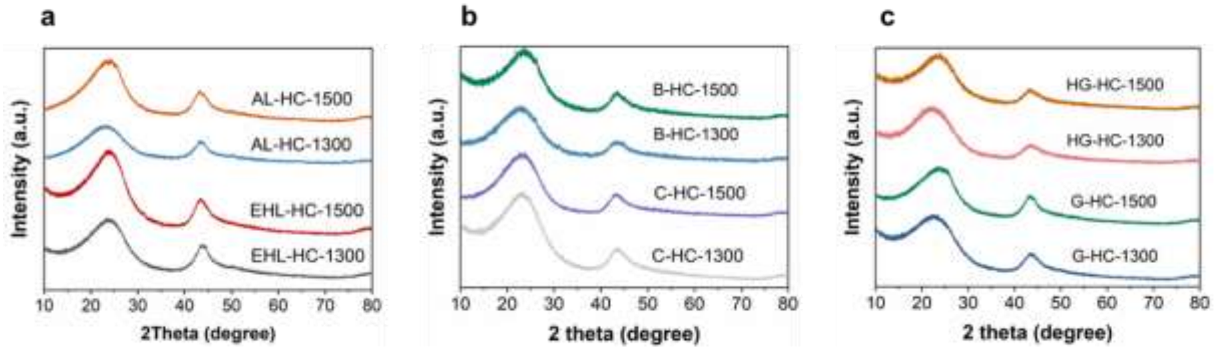

Figure S25. XRD pattern of (a) AL-HC-1300, AL-HC-1500, EHL-HC-1300, EHL-HC-1500, (b) B-HC-1300, B-HC-1500, C-HC-1300, C-HC-1500, (c) HG-HC-1300, HG-HC-1500, G-HC-1300, and G-HC-1500.

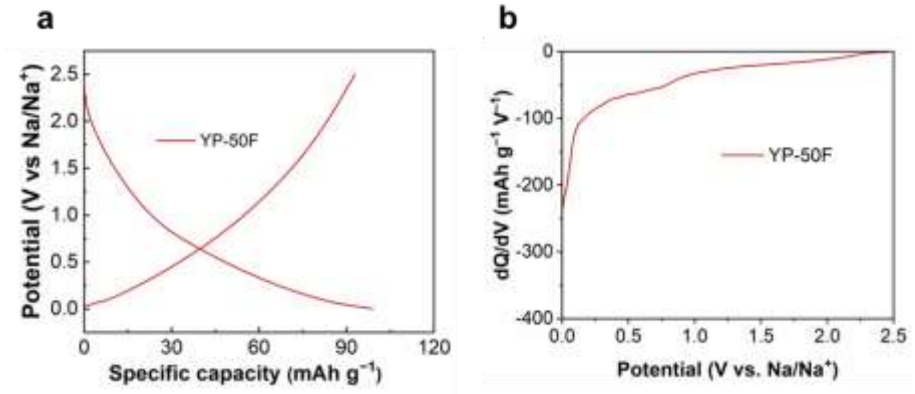

Figure S26. The (a) 2nd GCD curve and (b) dQ/dV curve of YP50F at a current density of 20 mA g^{-1}.

Note: The plateau-potential region was identified as the range where the derivative of specific capacity to potential (dQ/dV) was lower than –400 mAh g^{-1} V^{-1}, while the dQ/dV values higher than –400 mAh g^{-1} V^{-1} corresponded to the slope-potential region. The dQ/dV values of YP-50F porous carbon were all higher than –400 mAh g^{-1} V^{-1}. Therefore, the YP-50F porous carbon exhibited an absence of plateau-potential region. The plateau-potential capacity of YP-50F was 0 mAh g^{-1}. YP-50F porous carbon with open pores and high specific surface areas was incapable of storing sodium clusters.

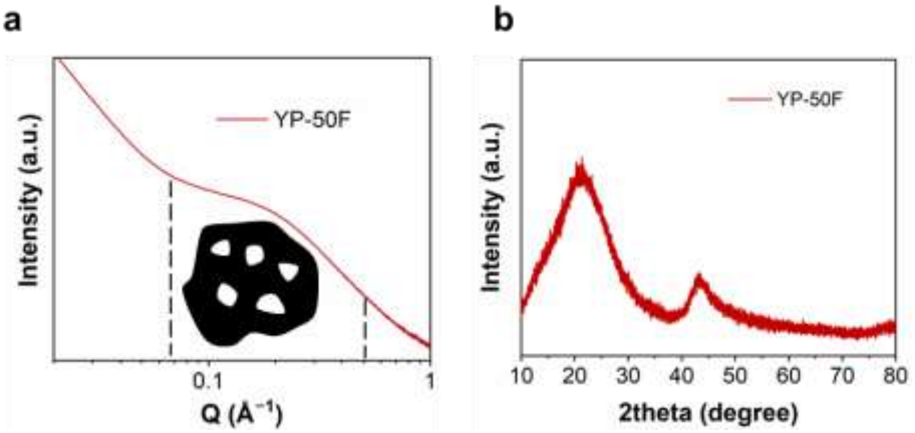

Figure S27. (a) SAXS and (b) XRD patterns of YP-50F porous carbon.

Note: From the SAXS patterns of YP-50F porous carbon, YP-50F porous carbon had abundant nanopores. The YP-50F porous carbon mainly has micropores and a high specific surface area of 1694 m^2 g^{-1}.[18] Although SAXS could estimate the nanopore volumes of YP-50F porous carbon, it could not differentiate the nanopores accessible and inaccessible to the solvent molecules of the electrolyte.

We probe the mechanism of sodium-ion storage behavior in YP-50F porous carbon with butanol pycnometry. The true density of the YP-50F porous carbon was tested as 2.199 g cm^{-3}, and the volume of nanopores inaccessible to the solvent molecules of the electrolyte was calculated as small as 0.002 cm^3 g^{-1} (Table S16). The nanopores of YP-50F porous carbon were almost open pores accessible to the solvent molecules of electrolytes, thus incapable of storing sodium clusters (**Figure S26**). It indicates that sodium clusters can hardly form in the open pores in this type of porous carbon. The underlying reason is that all the pores are open and large enough that they cannot host sodium clusters.

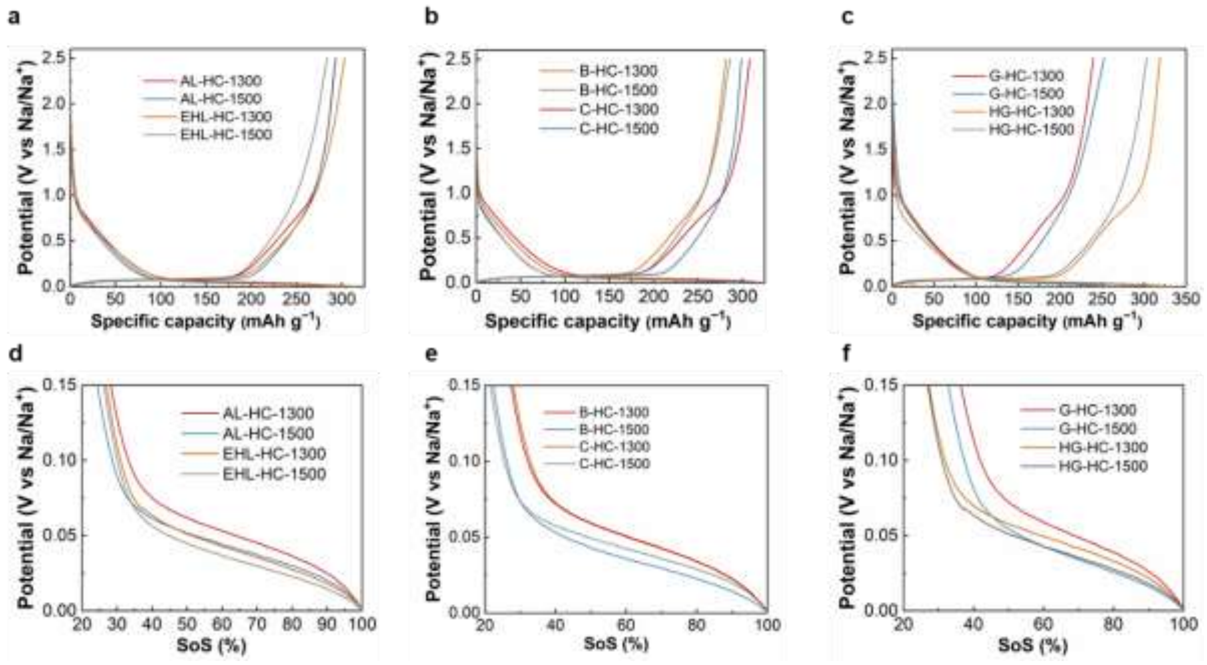

Figure S28. The 2nd GCD curves of (a, d) AL-HC-1300, AL-HC-1500, EHL-HC-1300, EHL-HC-1500, (b, e) B-HC-1300, B-HC-1500, C-HC-1300, C-HC-1500, (c, f) HG-HC-1300, HG-HC-1500, G-HC-1300, and G-HC-1500 at a current density of 20 mA g^{-1} .

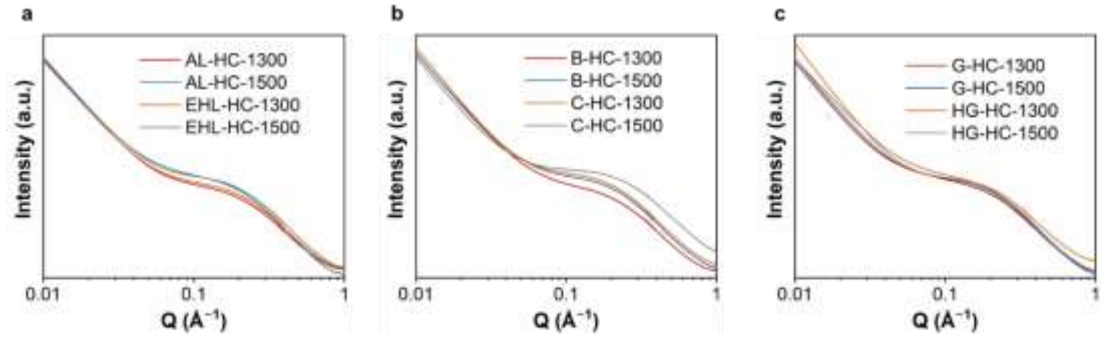

Figure S29. SAXS patterns of (a) AL-HC-1300, AL-HC-1500, EHL-HC-1300, EHL-HC-1500, (b) B-HC-1300, B-HC-1500, C-HC-1300, C-HC-1500, (c) HG-HC-1300, HG-HC-1500, G-HC-1300, and G-HC-1500.

References

1. Jian, W. B.; Qiu, X. Q.; Lai, Y. Y.; Yin, P. C.; Lin, J. X.; Zhang, W. L., Elucidation of the Mechanism of K-Ion Storage of Hard Carbon and Soft Carbon Anodes in Ether- and Ester-Based Electrolytes. *Advanced Energy Materials* **2023,** *13* (43), 2301303.
2. Wang, B.; Fitzpatrick, J. R.; Brookfield, A.; Fielding, A. J.; Reynolds, E.; Entwistle, J.; Tong, J.; Spencer, B. F.; Baldock, S.; Hunter, K.; Kavanagh, C. M.; Tapia-Ruiz, N., Electron paramagnetic resonance as a tool to determine the sodium charge storage mechanism of hard carbon. *Nature Communications* **2024,** *15* (1), 3013.
3. Zeng, J.; Bian, F.; Wang, J.; Li, X.; Wang, Y.; Tian, F.; Zhou, P., Performance on absolute scattering intensity calibration and protein molecular weight determination at BL16B1, a dedicated SAXS beamline at SSRF. *Journal of Synchrotron Radiation* **2017,** *24* (2), 509-520.
4. Saurel, D.; Segalini, J.; Jauregui, M.; Pendashteh, A.; Daffos, B.; Simon, P.; Casas-Cabanas, M., A SAXS outlook on disordered carbonaceous materials for electrochemical energy storage. *Energy Storage Materials* **2019,** *21*, 162-173.
5. Ilavsky, J.; Jemian, P. R., Irena: tool suite for modeling and analysis of small-angle scattering. *Journal of Applied Crystallography* **2009,** *42* (2), 347-353.
6. Kitsu Iglesias, L.; Antonio, E. N.; Martinez, T. D.; Zhang, L.; Zhuo, Z.; Weigand, S. J.; Guo, J.; Toney, M. F., Revealing the Sodium Storage Mechanisms in Hard Carbon Pores. *Advanced Energy Materials* **2023,** *13* (44), 2302171.
7. Stevens, D. A.; Dahn, J. R., An In Situ Small-Angle X-Ray Scattering Study of Sodium Insertion into a Nanoporous Carbon Anode Material within an Operating Electrochemical Cell. *J Electrochem Soc* **2000,** *147* (12), 4428-4431.
8. Morikawa, Y.; Nishimura, S.-i.; Hashimoto, R.-i.; Ohnuma, M.; Yamada, A., Mechanism of Sodium Storage in Hard Carbon: An X-Ray Scattering Analysis. *Advanced Energy Materials* **2020,** *10* (3), 1903176.
9. Cazorla-Amorós, D.; Alcañiz-Monge, J.; Linares-Solano, A., Characterization of Activated Carbon Fibers by CO2 Adsorption. *Langmuir* **1996,** *12* (11), 2820-2824.
10. Zhu, Y. W.; Murali, S.; Stoller, M. D.; Ganesh, K. J.; Cai, W. W.; Ferreira, P. J.; Pirkle, A.; Wallace, R. M.; Cychosz, K. A.; Thommes, M.; Su, D.; Stach, E. A.; Ruoff, R. S., Carbon-Based Supercapacitors Produced by Activation of Graphene. *Science* **2011,** *332* (6037), 1537-1541.
11. Zhang, S.-W.; Lv, W.; Luo, C.; You, C.-H.; Zhang, J.; Pan, Z.-Z.; Kang, F.-Y.; Yang, Q.-H., Commercial carbon molecular sieves as a high performance anode for sodium-ion batteries. *Energy Storage Materials* **2016,** *3*, 18-23.
12. Youn, Y.; Gao, B.; Kamiyama, A.; Kubota, K.; Komaba, S.; Tateyama, Y., Nanometer-size Na cluster formation in micropore of hard carbon as origin of higher-capacity Na-ion battery. *npj Computational Materials* **2021,** *7* (1), 48.
13. Li, Y.; Vasileiadis, A.; Zhou, Q.; Lu, Y.; Meng, Q.; Li, Y.; Ombrini, P.; Zhao, J.; Chen, Z.; Niu, Y.; Qi, X.; Xie, F.; van der Jagt, R.; Ganapathy, S.; Titirici, M.-M.; Li, H.; Chen, L.; Wagemaker, M.; Hu, Y.-S., Origin of fast charging in hard carbon anodes. *Nature Energy* **2024,** *9* (2), 134-142.
14. Li, Q.; Liu, X. S.; Tao, Y.; Huang, J. X.; Zhang, J.; Yang, C. P.; Zhang, Y. B.; Zhang, S. W.; Jia, Y. R.; Lin, Q. W.; Xiang, Y. X.; Cheng, J.; Lv, W.; Kang, F. Y.; Yang, Y.; Yang, Q. H., Sieving carbons promise practical anodes with extensible low-potential plateaus for sodium batteries. *National Science Review* **2022,** *9* (8), nwac084.
15. Liu, M.; Jiang, Z.; Wu, X.; Liu, F.; Li, W.; Meng, D.; Wei, A.; Nie, P.; Zhang, W.; Zheng, W., Reinventing the High‐rate Energy Storage of Hard Carbon: the Order‐degree Governs the Trade‐off of Desolvation‐Solid Electrolyte Interphase at Interfaces. *Angewandte Chemie International Edition* **2025,** *64* (17), e202425507.
16. Aniskevich, Y.; Yu, J. H.; Kim, J. Y.; Komaba, S.; Myung, S. T., Tracking Sodium

Cluster Dynamics in Hard Carbon with a Low Specific Surface Area for Sodium-Ion Batteries. *Advanced Energy Materials* **2024,** *14* (18), 2304300.
17. Ledwoch, D.; Komsiyska, L.; Hammer, E. M.; Smith, K.; Shearing, P. R.; Brett, D. J. L.; Kendrick, E., Determining the electrochemical transport parameters of sodium-ions in hard carbon composite electrodes. *Electrochimica Acta* **2022,** *401*, 139481.
18. Liu, X. Y.; Lyu, D.; Merlet, C.; Leesmith, M. J. A.; Hua, X.; Xu, Z.; Grey, C. P.; Forse, A. C., Structural disorder determines capacitance in nanoporous carbons. *Science* **2024,** *384* (6693), 321-325.
